# Boid inclusion body disease in *Boa constrictor*: Unraveling the myth of immunosuppression

**DOI:** 10.1016/j.isci.2026.117669

**Published:** 2026-09-22

**Authors:** Eva Dervas, Gábor Árpád Czirják, Eleni Michalopoulou, Annette Liesegang, Marilisa Novacco, Franz Schwarzenberger, Udo Hetzel, Jussi Hepojoki, Francesca Baggio, Anja Kipar

**Affiliations:** 1 Institute of Veterinary Pathology, Vetsuisse Faculty, University of Zurich, Zurich, Switzerland; 2 Department of Wildlife Diseases, Leibniz-Institute for Zoo and Wildlife Research, Berlin, Germany; 3 Institute for Animal Nutrition and Dietetics, Vetsuisse Faculty, University of Zurich, Zurich, Switzerland; 4 Clinical Laboratory, Vetsuisse Faculty, University of Zurich, Zurich, Switzerland; 5 Unit of Physiology, Pathophysiology and Experimental Endocrinology, Department of Biomedical Sciences, University of Veterinary Medicine Vienna, Vienna, Austria; 6 University of Helsinki, Faculty of Medicine, Medicum, Department of Virology, Helsinki, Finland

**Keywords:** immunosuppression, boid, phagocytosis, innate response, reptarenavirus, monocytes, azurophils

## Abstract

Boid inclusion body disease (BIBD) is an infectious disease of captive boid snakes of major relevance since the 1970s. It is caused by reptarenaviruses and characterized by intracytoplasmic inclusion bodies in most cell types. Affected snakes frequently develop secondary bacterial infections, suggesting virus-associated immunosuppression. To investigate this, we analyzed peripheral blood from 48 *Boa constrictor* with confirmed BIBD, three infected snakes without disease, and 84 reptarenavirus-negative snakes, assessing hematology, biochemistry, stress parameters, and immune function. A functional phagocytosis assay on peripheral blood revealed a significantly reduced proportion of phagocytic cells (Iba1^+^ monocytes/azurophils) in BIBD-positive and reptarenavirus-infected snakes compared to uninfected animals, indicating impaired phagocytic capacity. In addition, BIBD-positive snakes showed reduced cholesterol and total immunoglobulin concentrations, while virus-specific IgY and IgM levels were elevated. These findings indicate selective impairment of innate and humoral immune components, providing mechanistic evidence for increased susceptibility to secondary bacterial infections in BIBD.

## Highlights

• BIBD is associated with impaired monocyte/macrophage function
• Impaired phagocytic activity indicates impairment of the innate immune response in BIBD
• BIBD is associated with a severe decrease in cholesterol levels

## Graphical abstract

**Figure:**
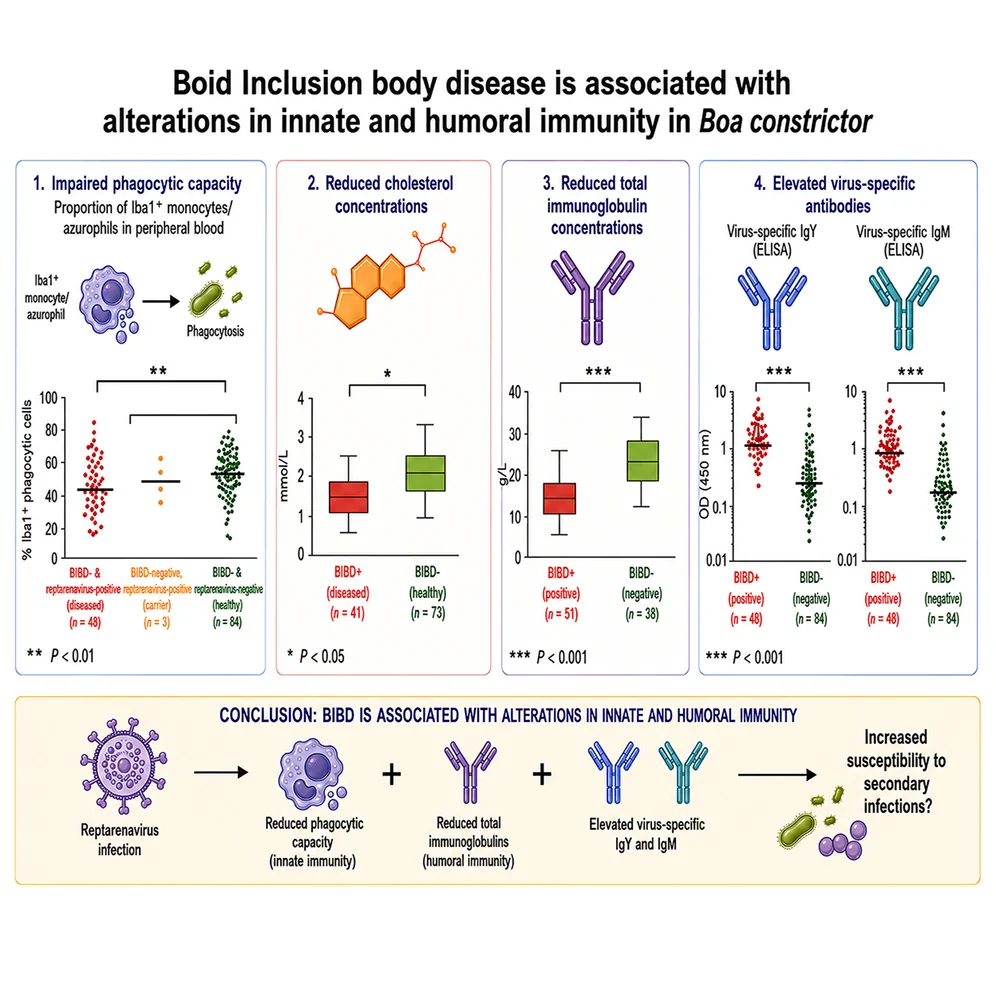

## Teaser

Virology; Veterinary medicine; Microbiology

## Introduction

Since the late 1970s, boid inclusion body disease (BIBD) has consistently been one of the most important infectious diseases of captive boid snakes worldwide. BIBD is a viral disease, its causative agents are genetically highly divergent reptarenaviruses, as identified by several groups in the early 2010s.^1^^,^^2^^,^^3^ Pathomorphologically, BIBD is characterized by intracytoplasmic inclusion bodies (IB) in a large variety of cell types in the affected snake.^1^^,^^2^^,^^4^^,^^5^^,^^6^ These IB led to the name of the disease as they are its only consistent feature and hence the diagnostic hallmark. IB, which are apparent as variable sized eosinophilic to amphophilic, intracytoplasmic structures, are primarily composed of the ∼68 kDa reptarenavirus nucleoprotein (NP).^2^ Recent studies have shown that boas can be reptarenavirus-infected and viremic^7^ without or prior to IB formation; it is not yet clear whether the development of IB reflects a pathological process that is correlated with functional alterations. Reptarenaviruses can be transmitted both vertically and horizontally. Vertical transmission has been demonstrated in *Boa constrictor*,^8^ whereas horizontal transmission is suspected to occur in captivity, and likely; nonetheless, the precise transmission routes are still unknown.^8^^,^^9^ Snakes with BIBD frequently carry multiple genetically divergent reptarenaviruses, which can be considered as further evidence of potentially multiple transmission events.^10^ The effect of co-infection with multiple reptarenavirus species, and of concurrent infections with other viruses, as well as the incubation period, and possible risk factors contributing to the onset of BIBD are currently unknown.^8^^,^^9^^,^^10^^,^^11^

When BIBD was first described, in the United States of America, the disease was mainly described as a neurological disorder that affected multiple species of boid snakes in private and zoological collections,^4^^,^^12^ e.g., boa constrictors (*B. constrictor*), emerald tree boas (*Corallus caninus*), and Burmese pythons (*Python molurus bivittatus*). Among these, the *B. constrictor* is of particular relevance, as it is not only one of the most commonly traded, kept, and bred snake species in captivity, but also most frequently affected by the disease.^13^ At the time, clinical signs primarily involved the central nervous system (CNS) and comprised, e.g., torticollis, disequilibrium, head tremors, regurgitation, opisthotonos (“stargazing”), disturbance of equilibrium, an inability of the snakes to right themselves when placed in dorsal recumbency, as well as flaccid paralysis.^4^ However, in the authors’ experience, over the last decades clinical CNS signs have become less frequent in boas with BIBD. Instead, non-specific clinical signs, such as apathy, anorexia, regurgitation and weight loss have become more frequent, often in association with bacterial, fungal and protozoal infections.^5^^,^^6^^,^^12^^,^^14^^,^^15^ In rare cases, neurologic disease is still observed but it may show a more subtle clinical manifestation, with disequilibrium, regurgitation, head tremor, or loss of muscular tonus (personal observation). The frequent association of BIBD with bacterial and/or parasitic co-infections in boas has most likely led to the widely accepted hypothesis that BIBD associates with immunosuppression.^12^^,^^16^ A few reports have described neoplastic processes in snakes with BIBD.^17^^,^^18^^,^^19^^,^^20^ While this could indicate a potential association between the two entities, it could also represent a pure coincidence in light of the relatively high prevalence of neoplastic processes and particularly lymphoma, in snakes.^21^ Indeed, immunosuppression is a feature of some mammalian arenavirus infections. For example, infection of mice with lymphocytic choriomeningitis virus (LCMV), a mammarenavirus, leads to immunosuppression due to functional impairment of the antigen presenting cells (APCs) and suppression of the IFN-I signaling pathway by direct binding of viral proteins to effector proteins.^22^^,^^23^

Despite claims in association with BIBD, an immunosuppressive effect has so far not been confirmed for reptarenaviruses in their snake hosts.^12^^,^^16^ Similarly, little research has been conducted on the immune response of infected snakes to the viral infection. In a previous study, we developed an ELISA to detect NP-specific IgY and IgM antibodies in *B. constrictor*, aiming to gain insight into the immune response of this species to reptarenaviruses. This revealed a negative correlation between BIBD and the presence of IgY antibodies against viral NP^24^ and showed that individuals with BIBD have lower anti-NP specific IgY levels than reptarenavirus-infected animals without IB.^7^ Furthermore, naturally reptarenavirus-infected animals without BIBD demonstrated higher levels of reptarenavirus neutralizing antibodies than experimentally infected animals without BIBD.^7^ In a recent morphological study investigating potential alterations in the hemolymphatic tissues of boa constrictors with BIBD, we demonstrated infection of various hematopoietic precursor cell lines in the bone marrow in association with the disease.^25^ This finding suggests that the bone marrow serves as a niche for viral persistence and could contribute to or mediate (intermittent or recurrent) viremia. We also observed a significantly increased cellularity and a granulomatous response in the spleens of snakes with BIBD, indicating that reptarenavirus infection might be linked to alterations in the splenic microenvironment; yet the functional consequences of this reaction warrant further analyses.^25^

Knowledge of the reptilian immune response is overall still very limited. However, it is clear that, like in other animal classes, the reptilian immune system comprises both an innate and adaptive immune response.^26^^,^^27^^,^^28^Reptiles rely more heavily on their innate immune response than mammals, which includes physical barriers as well as cellular and humoral components such as heterophils, monocytes/macrophages, complement factors, lysozyme, antimicrobial peptides, and natural antibodies.^26^^,^^28^ The adaptive immune system is mediated by B and T cells and involves antigen-specific responses, including immunoglobulin production (primarily IgM and IgY) and cell-mediated immunity; however, these responses tend to be slower and less robust than in mammals, even upon repeated exposure to pathogens.^26^^,^^28^^,^^29^ Additionally, their poikilothermic nature makes reptiles notably distinct from other animal classes; as they cannot regulate their body temperature internally, resulting in immune responses that differ significantly depending on the environmental temperatures.^30^^,^^31^^,^^32^^,^^33^^,^^34^^,^^35^^,^^36^ These factors highlight the challenges of studying viral infections in reptiles and underscore the need for more targeted research to gain a deeper understanding of the immunopathological processes in reptilian species. Reptarenavirus infection and BIBD in boa constrictors provides a promising natural model to study the latter, as its long-term nature likely poses various continuous challenges to both the innate and adaptive immune system.

We hypothesize that BIBD compromises both innate and adaptive immune functions in *B. constrictor* and expect these to be reflected in alterations of hematological and biochemical parameters, impaired antibody responses, and increased stress hormone levels. To address this hypothesis, we conducted a comprehensive analysis of several blood parameters, including hematological and biochemical analyses and corticosterone measurements. Additionally, we performed several assays targeting various components of both the innate and adaptive immune response. The results demonstrate that a range of immune functions are affected by BIBD; this can now direct the design of more targeted investigations to elucidate the pathomechanisms mediating the disease.

## Results

### Study population, BIBD and reptarenavirus infection status, and clinical data

The study population comprised 133 captive *B. constrictor* from different breeding and private collections, including a reptile shelter. BIBD was diagnosed in 49 animals, based on the presence of IB in cells of the peripheral blood (Figure S1A). Of these, 41 animals had been subjected to a postmortem examination. In all, the histological examination, combined with immunohistology for reptarenavirus nucleoprotein,^2^ confirmed the presence of IB also in parenchymal cells in inner organs (e.g., brain, liver, kidneys (Figures S1B–S1D and Table S1), as previously described.^8^^,^^25^^,^^37^

To confirm/exclude reptarenavirus infection and to detect carriers, i.e., animals that were reptarenavirus-infected but did not suffer from BIBD, the peripheral blood of all animals was examined by the multiplex RT-PCR for reptarenavirus S segments.^38^ This confirmed reptarenavirus infection in all BIBD-positive (BIBD+) animals (*n* = 49) and identified three infected animals without evidence of IB (BIBD−/vRNA+; A21, E7, and G3; Table S1); these animals are hence considered as carriers.^39^ All remaining BIBD-negative animals tested negative (*n* = 83) in the multiplex RT-PCR, i.e., did not yield an RT-PCR product of the appropriate base pair length in the agarose gel electrophoresis and are therefore considered as uninfected (BIBD−/vRNA−).

The age was known for 123 animals and ranged from 3 months to 25 years, with a mean age of 5 years; in BIBD−/vRNA− snakes, it ranged from 3 months to 20 years; 33 animals were female, 50 male, in 15 juveniles the sex could not be determined due to lack of differentiation of the gonads. In BIBD+ snakes, the age ranged from 1 year to 20 years; 27 animals were female, and 22 were male. The three BIBD−/vRNA+ snakes were 2, 4, and 11 years old and two male and one female, respectively. The ten individuals whose age was not known were classified as adult (≥3 years) based on their size and length and the history provided by the owners.

Similar to our earlier study^40^ to allow statistical analysis of age-related differences, two age groups (“young” and “adult”) were defined, “young” referring to animals <3 years of age (prior to definite sexual maturity and reproduction;^41^), and “adult” to animals ≥3 years of age (sexually mature, reproducing animals).

According to their clinical history, two individuals had lost weight (B11 and B14), two had shown diarrhea (B16 and B17), and one had shown respiratory distress (A23). All these animals were BIBD+ (Table S1). Four animals, C27 (BIBD−/vRNA−), C30, B4, and B5 (all BIBD+), were in a state of ecdysis (shedding). All remaining animals had not shown any clinical signs prior to euthanasia or blood sampling.

BIBD and reptarenavirus infection are linked to alterations in phagocytic cells and phagocytic function that are independent of age- or sex-associated variations.

#### Hematological parameters

Hematological data were collected from 88 animals. BIBD− animals (*n* = 47; vRNA−: *n* = 44; vRNA+: *n* = 3) showed a significantly higher percentage of monocytes (geometric mean = 2.4%, CI = 2.02–2.86) than BIBD+ animals (*n* = 41; geometric mean = 1.62%, CI = 1.2–2.17, t = 2.439, df = 80, *p* < 0.05) (Figure 1A and Table S2). Interestingly, this significant difference was not detected when vRNA+ (*n* = 44) and vRNA− (*n* = 47) snakes were compared (Table S2).

**Figure 1:**
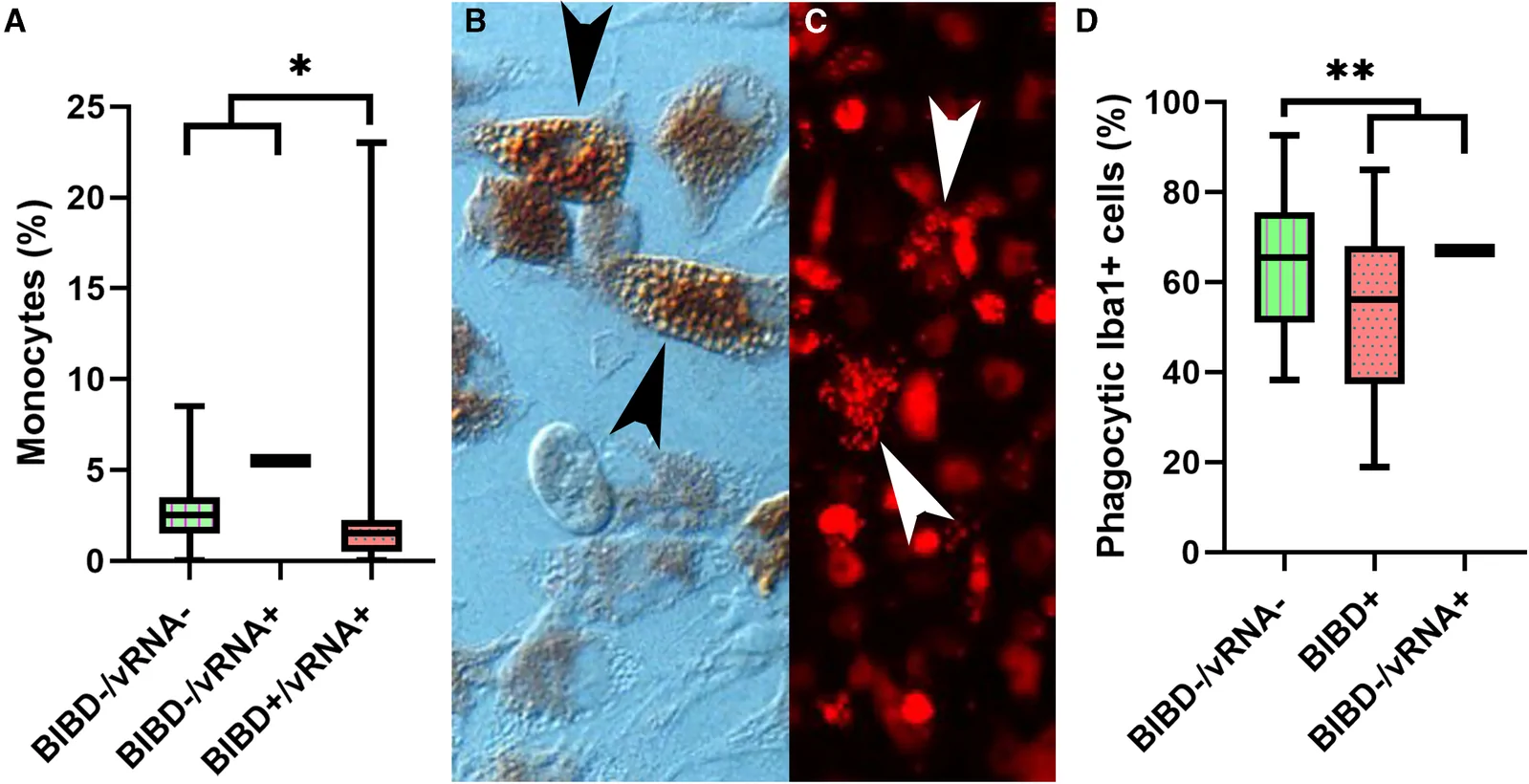
Iba1 positive cells and phagocytosis in the peripheral blood (A) BIBD+ animals (*n* = 41) have a significantly lower percentage of circulating monocytes compared to BIBD−/vRNA− animals (*n* = 47) and carrier animals (*n* = 3) (BIBD−/vRNA+) (t = 2.439, df = 80). (B) Peripheral blood monocytes and/or azurophils (Iba1+) with intracytoplasmic fluorescent beads (arrowheads). Brightfield with phase contrast; animal C30. (C) Intracytoplasmic fluorescent beads in monocytes (arrowheads). Tetramethylrhodamine Isothiocyanate (TRITC) filter; animal H3. (D) BIBD+ animals (*n* = 48) have a significantly lower percentage of phagocytic Iba1+ cells compared to BIBD−/vRNA− animals (*n* = 33) and one carrier animal (BIBD−/vRNA+) (t = 3.3725, df = 80). BIBD, boid inclusion body disease; vRAV, virus RNA of reptarenavirus. Data in (A) and (D) are presented as box-and-whisker plots showing the median and interquartile range (IQR), with whiskers extending from the minimum to the maximum value. Statistical significance was assessed using a two-sample *t* test. (∗) indicates a *p* value <0.05, (∗∗) a *p* value <0.01 and (∗∗∗) a *p* value <0.001.

After controlling for the effect of age, sex, and clustering by breeder via multiple regression, a significant association was also found between BIBD and the number of heterophils/μL (R^2^ = 0.1778, F(3,5) = 161, *p* < 0.05) and the number of monocytes/μL (R^2^ = 0.0438, F(3,5) = 6.95, *p* < 0.01); both were lower in BIBD+ animals (Figure 1A and Table S6). A significant association was found between reptarenavirus infection and the following hematological parameters: number of heterophils/μL (R^2^ = 0.1867, F(3,5) = 188.86, *p* < 0.05), number of eosinophils/μL (R^2^ = 0.0733, F(3,5) = 27.99, *p* < 0.01), number of monocytes/μL (R^2^ = 0.0438, F(3,5) = 6.95, *p* < 0.01) and mean cell hemoglobin concentration (MCHC) (R^2^ = 0.0520, F(3,5) = 65.08, *p* < 0.01). The number of heterophils, eosinophils and monocytes was lower and the MCHC was higher in vRNA+ animals (Table S5).

However, the percentage and number of heterophils and lymphocytes, the percentage of azurophils and the hematocrit showed a significant association with age regardless of BIBD and reptarenavirus infection status. More specifically, the percentage and number of heterophils (R^2^ = 0.02460, F(3,5) = 34.39, *p* < 0.01 and R^2^ = 0.1867, F(3,5) = 188.86, *p* < 0.05, respectively), the percentage of azurophils (R^2^ = 0.0820, F(3,5) = 8.72, *p* < 0.01) and the hematocrit (R^2^ = 0.0828, F(3,5) = 10.99, *p* < 0.05) were positively correlated with age. In contrast, the percentage and number of lymphocytes showed a negative correlation with age (R^2^ = 0.2110, F(3,5) = 19.44, *p* < 0.01 and R^2^ = 0.1078, F(3,5) = 3.42, *p* < 0.05, respectively) (Tables S5 and S6). BIBD+/vRNA+ snakes with clinical signs did not differ significantly from BIBD+/vRNA+ snakes without clinical signs or from BIBD−/vRNA− snakes in hematological parameters (all Dunn’s adjusted *p* > 0.05) (Table S7).

#### Phagocytosis assay

The phagocytosis assay was performed on the buffy coat of 82 animals (33 BIBD−/vRNA−, 48 BIBD+ and 1 BIBD−/vRNA+). As shown in our previous study, the monocytes of the *B. constrictor* are Iba1-positive and represent a relatively small fraction of cells in the peripheral blood.^33^ We also characterized a second cell type, azurophils, which possess morphological and immunohistochemical properties (such as Iba1 positivity) similar to those of monocytes and are capable of phagocytosis.^40^ Hence, we were confident to categorize all Iba1-positive phagocytic cells identified in the phagocytosis assay as monocytes or azurophils. These Iba1-positive cells were round to polygonal and generally presented as individual cells with abundant intracytoplasmic fluorescent beads, indicating their high phagocytic activity (Figures 2B and 2C). In contrast, Iba1-negative phagocytic cells exhibited a variable morphology; some were round to oval in shape with abundant cytoplasm while others were smaller with a limited amount of cytoplasm. They could not be characterized further within the scope of the immunofluorescent examination.

**Figure 2:**
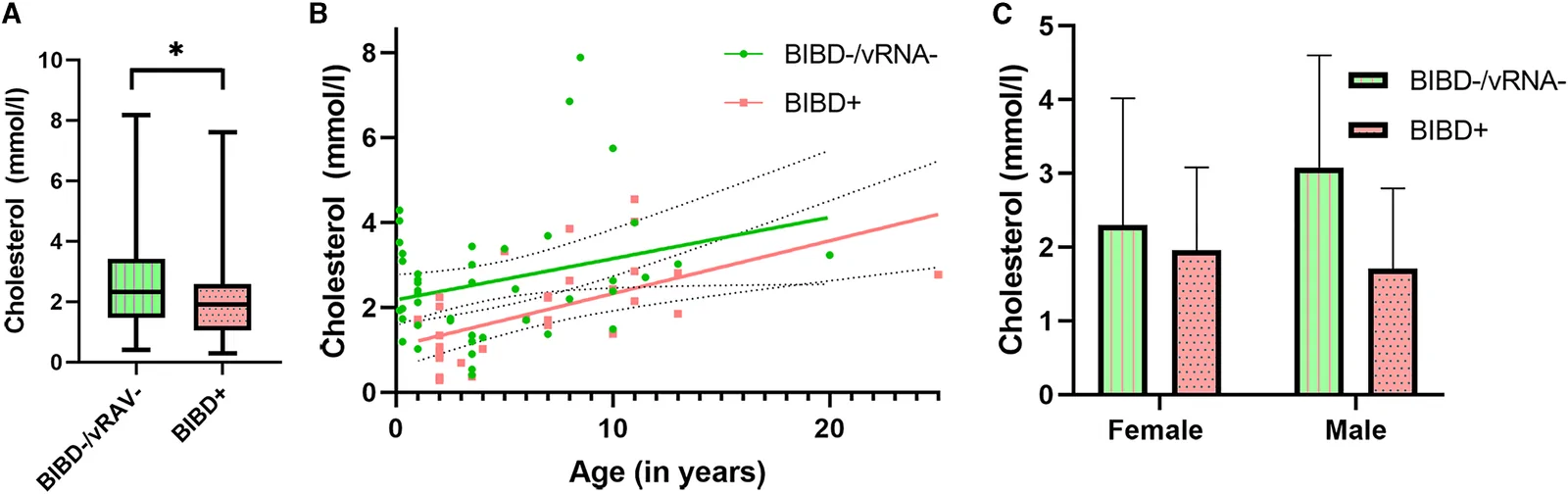
Cholesterol levels in the peripheral blood BIBD−/vRNA− animals (*n* = 71) show significantly higher cholesterol levels than BIBD+ animals (A; t = −2.41, df = 112, *n* = 41). Cholesterol levels show a significant association with age (B) and sex (C) (R^2^ = 0.2370, F(3,9) = 2.44, *p* < 0.01 for both parameters). BIBD, boid inclusion body disease; vRAV, virus RNA of reptarenavirus. Data are presented as box-and-whisker plots showing the median and interquartile range (IQR), with whiskers extending from the minimum to the maximum value. In (A), statistical significance was assessed using a two-sample *t* test. In (B) and (C), multiple linear regression was used to assess the association of cholesterol levels with age and sex. (∗) indicates a *p* value <0.05, (∗∗) a *p* value <0.01 and (∗∗∗) a *p* value <0.001.

The percentage of phagocytic Iba1+ cells was significantly higher in BIBD−/vRNA− animals (mean = 64.36, CI = 60.14–68.57) than in BIBD+ animals (mean = 52.2, CI = 45.88–58.52), with t = 3.37, df = 80, *p* < 0.01 (Figure 1D), and, similarly, significantly higher in vRNA− animals (mean = 64.93, CI = 60.53–69.32) than in vRNA+ animals (mean = 52.48, CI = 46.65–58.32), with t = −3.52, df = 80, *p* < 0.001 (Table S3). After controlling for the effect of age, sex and clustering by breeder, the multiple regression analysis showed a significant association between reptarenavirus infection and BIBD and the percentage of phagocytic Iba1+ cells (R^2^ = 0.2489, F(3,5) = 61.08, *p* < 0.01 and R^2^ = 0.2503, F(3,6) = 83.22 respectively, *p* < 0.01) (Table S5). The percentage of phagocytic Iba1+ cells did not differ significantly in BIBD+/vRNA+ snakes with clinical signs from the values in BIBD+/vRNA+ snakes without clinical signs or BIBD−/vRNA− snakes (Dunn’s adjusted *p* > 0.05) (Table S7).

Other components of the innate immune system and the stress response remain unaltered with BIBD or reptarenavirus infection.

#### Lysozyme concentration

Lysozyme concentrations, determined in the plasma of 79 animals (BIBD+: *n* = 35; BIBD−/vRNA−: *n* = 43; BIBD−/vRNA+: *n* = 1) did not vary significantly with BIBD or infection status (Table S3). However, they showed a significant association with sex, with female animals exhibiting higher levels (R^2^ = 0.1501, F(3,5) = 6.77, *p* < 0.05) (Tables S5 and S6).

#### Hemagglutination/hemolysis (HA/HL)

The Nab and complement titers were measured in 78 individuals (BIBD+: *n* = 35; BIBD−/vRNA−: *n* = 43; BIBD−/vRNA+: *n* = 1). The plasma samples of all animals showed clear agglutination and hemolysis with the sheep red blood cells, indicating an effective interaction with the cell membrane. However, the HA/HL titers did not vary significantly with BIBD and infection status and did not show a significant association with sex and age (Tables S3, S5, and S6).

#### Stress parameters

The stress parameters (corticosterone and glucose levels, heterophil/lymphocyte ratio) did not vary significantly with BIBD and infection status and did not show a significant association with sex and age (Tables S3, S5, and S6).

BIBD+/vRNA+ snakes with clinical signs did not differ significantly from BIBD+/vRNA+ snakes without clinical signs or BIBD−/vRNA− snakes with regards to lysozyme concentration, Nab and complement titers and stress parameters (all Dunn’s adjusted *p* > 0.05) (Table S7).

BIBD and reptarenavirus infection are associated with lower cholesterol levels, independent of age- and sex-related variations.

#### Biochemical parameters

Biochemical analysis of total protein (TP), albumin, cholesterol, uric acid, glucose, urea, aspartate transaminase (AST), and lactate dehydrogenase (LDH) was performed in 114 animals. BIBD+ (*n* = 41) animals had significantly lower cholesterol levels (geometric mean = 1.65%, CI = 1.3–2.1), with t = −2.41, df = 112, *p* < 0.05 than BIBD− animals (vRNA−: *n* = 71; vRNA+: *n* = 2; geometric mean = 2.29%, CI = 1.97–2.67) (Figure 2A and Table S4). The same results were obtained when animals were grouped according to their infection status: vRNA+ animals (*n* = 44) had significantly lower cholesterol levels (geometric mean = 1.68%, CI = 1.34–2.11), with t = −2.53, df = 106, *p* < 0.05) than vRNA− animals (*n* = 73; geometric mean = 2.38%, CI = 2–2.8) (Table S4).

After controlling for the effect of age, sex, and clustering by breeder, via multiple regression, a statistically significant association was found between reptarenavirus infection and BIBD and cholesterol levels. These were lower with reptarenavirus infection and BIBD, with R2 = 0.2786, F(3,9) = 27.03, *p* < 0.05 and R2 = 0.2370, F(3,9) = 43.32, *p* < 0.05, respectively (Tables S5 and S6). Cholesterol levels also showed a significant association with age and sex (R2 = 0.2370, F(3,9) = 2.44, *p* < 0.01 for both parameters; Figures 2B and 2C). Regardless of BIBD and infection status, they increased with age, as demonstrated in Figure 2B.

AST levels also showed a significant association with sex but not with disease and/or infection status, with male animals showing higher levels (R^2^ = = 0.051, F(3,5) = 3.98, *p* < 0.05) (Tables S5 and S6).

All remaining biochemical parameters (TP, albumin, uric acid, glucose, urea, and LDH) did not vary significantly with BIBD and infection status (Tables S4, S5 and S6). The biochemical parameters did not differ significantly between BIBD+/vRNA+ snakes with clinical signs, BIBD+/vRNA+ snakes without clinical signs and BIBD−/vRNA− snakes (all Dunn’s adjusted *p* > 0.05) (Table S7).

BIBD and reptarenavirus infection are associated with lower total immunoglobulin concentrations but higher virus-specific antibody levels.

#### Total immunoglobulin

BIBD− animals (vRNA−: *n* = 35; vRNA+: *n* = 3) had significantly higher plasma total immunoglobulin concentrations (geometric mean = 16.97%, CI = 14.67–19.63) than BIBD+ animals (*n* = 51; geometric mean = 11.23%, CI = 9.27–13.6), with t = 3.5, df = 88, *p* < 0.001 (Figure 3A). When only the infection status was considered, vRNA− animals were found to have higher immunoglobulin concentrations (geometric mean = 17.1%, CI = 14.65–19.87) than vRNA+ animals (geometric mean = 11.67%, CI = 9.69–14.1), with t = 3.2, df = 87, *p* < 0.01) (Table S3).

**Figure 3:**
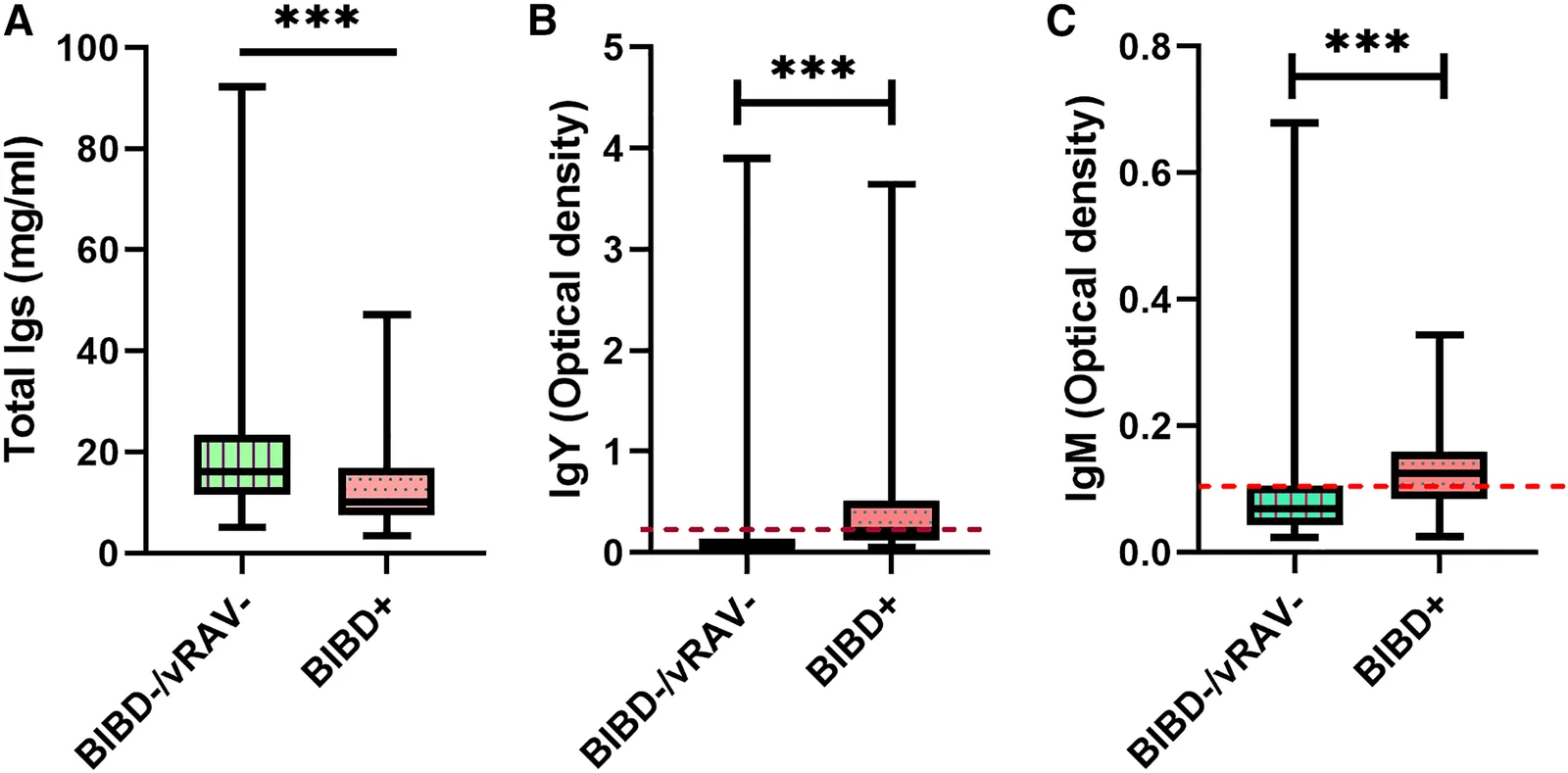
Humoral immune response (A) BIBD−/vRNA− animals (*n* = 35) show higher total immunoglobulin concentrations than BIBD+ animals (t = 3.5, df = 88, *n* = 51). (B) BIBD−/vRNA− animals (*n* = 60) show lower anti-UGV-1 IgY OD values than BIBD+ animals (z = −4.1, *n* = 35). The red line indicates the cut-off value set at 0.22. (C) BIBD−/vRNA− animals (*n* = 60) show lower anti-UGV-1 IgM OD values than BIBD+ animals (z = −3.75, *n* = 35). The red line indicates the cut-off value set at 0.086. BIBD, boid inclusion body disease; vRAV, virus RNA of reptarenavirus; Ig, immunoglobulin. Data are presented as box-and-whisker plots showing the median and interquartile range (IQR). Whiskers extending from the minimum to the maximum value. In (A), statistical significance was assessed using a two-sample *t* test for normally distributed data. In (B) and (C), statistical significance was assessed using the Mann-Whitney U test for non-normally distributed data. ∗*p* < 0.05, ∗∗*p* < 0.01, and ∗*p* < 0.001. (∗) indicates a *p* value <0.05, (∗∗) a *p* value <0.01 and (∗∗∗) a *p* value <0.001.

The significant association between reptarenavirus infection/BIBD and the total immunoglobulin concentration was also confirmed by multiple regression analysis, after controlling for the effect of age, sex, and clustering by breeder (R^2^ = 0.0893, F(3,8) = 13.63, *p* < 0.05 and R^2^ = 0.0999, F(3,5) = 41.69 respectively, *p* < 0.05) (Table S5).

#### Anti-reptarenavirus IgM and IgY levels

The ELISA OD values for anti-UGV-1-IgM and IgY levels were determined in the plasma of 96 animals (BIBD+: *n* = 35; BIBD−/vRNA−: *n* = 60; BIBD−/vRNA+: *n* = 1). BIBD+ animals were found to have higher UGV-1 IgY (median = 0.222, CI = 0.17–0.36) and IgM (median = 0.125, CI = 0.098–0.149) ELISA OD values than BIBD− animals (IgY: median = 0.0528, CI = 0.04–0.089; IgM: median = 0.0691, CI = 0.057–0.081), with z = −4.1, *p* < 0.0001 (Figure 3B and Table S4) and z = -3.75, *p* < 0.0001 (Figure 3C and Table S3), respectively.

vRNA+ animals were also found to have higher UGV-1 IgY (median = 0.222, CI = 0.17–0.36) and IgM (median = 0.125, CI = 0.098–0.149) ELISA OD values than vRNA− animals (IgY: median = 0.0528, CI = 0.04–0.089; IgM: median = 0.0691, CI = 0.057–0.081), with z = −3.89, *p* < 0.0001 (Figure 3B and Table S3) and z = −3.68 and *p* = 0. 0002 (Table S3), respectively.

#### ELISA cut-off points

In addition to the absolute OD values, the raw ELISA data were first corrected for background. Because background-corrected values can still include nonspecific signals or plate-to-plate variation, the data were further analyzed using a cut-off value, following the protocol of a previous study.^24^ The background corrected raw ELISA data with cut-off values are presented in Figures 3B and 3C. Of the 35 BIBD+ snakes, 27 (77.1%) tested positive for anti-UGV-1 antibodies. Seventeen (48.6%) were anti-UGV-1 IgY positive, and 24 (68.6%) IgM positive, 14 (40%) were positive for both. Of the 60 BIBD−/vRNA− snakes, 22 (36.7%) carried anti-UGV-1 antibodies; 14 (23.3%) had anti-UGV-1 IgY and 19 (31.7%) IgM antibodies, 11 animals (18.3%) showed both IgY and IgM. Information on the presence of anti-UGV-1 IgY and IgM antibodies in each animal is provided in Table S1.

#### T cell stimulation

The leukocyte blast transformation test was performed on the plasma of 46 animals (BIBD+: *n* = 13; BIBD−/vRNA−: *n* = 30; BIBD−/vRNA+: *n* = 3). This revealed a statistically significant difference between BIBD− and BIBD+ animals in the control (non-PHA stimulated) groups. Specifically, BIBD−, non-PHA stimulated animals (vRNA−: 30; vRNA+: 3) showed a higher blast transformation (stimulation) index (mean = 20.27, CI = 16.72–23–82) than BIBD+, non-PHA stimulated animals (*n* = 46; mean = 13.44, CI = 10.8–16.1, with t = 2.36, df = 44, *p* < 0.05) (Table S3). Statistically significant differences were not observed between groups after PHA stimulation. In the multiple regression analysis, after controlling for the effect of age, sex, and clustering by breeder, a statistically significant association was found between reptarenavirus infection and BIBD and the blast transformation (stimulation) index of the non-PHA stimulated control group (R^2^ = 0.2335, F(3,6) = 6.49, *p* < 0.05 and R^2^ = 0.2600, F(3,6) = 5.31, *p* < 0.01, respectively) (Table S5). BIBD+/vRNA+ snakes with clinical signs did not differ significantly from BIBD+/vRNA+ snakes without clinical signs or BIBD−/vRNA− snakes in their total immunoglobulin levels and anti-reptarenavirus IgM and IgY levels, and in the leukocyte blast transformation test (all Dunn’s adjusted *p* > 0.05) (Table S7).

## Discussion

The present study aimed to gain information on the potential effect of BIBD on hematological, biochemical, and stress parameters, as well as immune functions of the *B. constrictor*, an area for which solid data did not yet exist. So far, only one study, published in 1994, has addressed hematological and biochemical changes in BIBD.^4^ . The authors examined the blood values of 21 boid snakes with BIBD, confirmed by the presence of intracytoplasmic IB in tissue parenchymal cells, e.g., in epithelial cells (pancreatic acinar cells, thyroid cells, etc.) and hepatocytes.^4^ The diseased snakes were classified as “acutely infected” and “chronically infected,” although the basis for this categorization was not specified. Of note, the study was retrospective and conducted prior to the identification of reptarenaviruses as the causative agents of BIBD. In any case, leukocytosis, relative lymphocytosis, lower protein and globulin levels and significantly higher aspartate aminotransferase (AST) levels were reported in the “acutely infected” snakes vs*.* the “chronically infected” snakes; as another caveat, disease free control animals were not included.^4^

The present study assessed a wide range of hematological (absolute and relative leukocyte counts, hematocrit, hemoglobin, MCHC) and biochemical parameters (cholesterol, TP, albumin, urea, uric acid, AST, and LDH) in boa constrictors with BIBD and confirmed reptarenavirus infection, as well as in boas without BIBD in which the multiplex-RT-PCR had either identified reptarenavirus infection or had confirmed that the animals were not infected. Surprisingly, apart from a significant reduction in circulating monocytes (discussed later) and significantly lower cholesterol levels, BIBD (and reptarenavirus infection) appeared not to affect the examined blood parameters. There was a trend for hepatic parameters, such as AST, albumin and TP, to be elevated in BIBD+ animals; however, the difference was not statistically significant. The latter is likely not a consequence of insufficient sample size, as we examined a large number of animals (between 92 and 114) with a broad age range (3 months to 25 years). However, the time point post reptarenavirus infection and the duration of the disease (i.e., the onset of BIBD development) were not known, and none of the animals with BIBD had shown severe clinical signs, so it cannot be excluded that we did not catch the optimal time point post infection to spot any significant hematological alterations.

The fact that BIBD+ snakes exhibited significantly reduced blood cholesterol levels is noteworthy, particularly in light of studies on various infectious diseases in humans. For instance, chronic human immunodeficiency virus (HIV) infections are associated with lower levels of lipid metabolites (cholesterol, lipoproteins, high-density-lipids etc.).^42^ In human pulmonary tuberculosis, lower serum cholesterol levels directly correlate with the severity of infection, and in patients with severe sepsis, they are also predictive of mortality.^43^ Overall, several factors are known to contribute to the development of hypocholesterolaemia in mammals, including reduced body weight, low dietary lipid intake, impaired or diminished hepatic function (e.g., due to underlying disease), and chronic inflammatory processes. Given that the snake population examined in this study originated from multiple collections and was therefore subject to varying husbandry practices, dietary composition is unlikely to be the primary driver of the observed reduction in plasma cholesterol concentrations. Moreover, the BIBD+ boas did not demonstrate a statistically significant reduction in body weight. This contrasts with our previous findings in female boas affected by BIBD.^24^ Taking into account the generally elevated (albeit non-significant) hepatic enzyme activities that we observed in BIBD+ snakes, impaired hepatocyte function might be the underlying pathomechanism. In a recent study, we demonstrated that reptarenavirus NP, the main protein component of the IB, accumulates within the mitochondria of infected cells, suggesting that it may affect mitochondrial metabolism and thereby cellular homeostasis.^38^ IB formation in the majority of hepatocytes is a key morphological feature of BIBD in boas. Given the role of hepatocyte mitochondria in lipid metabolism,^44^^,^^45^ viral-induced mitochondrial dysfunction may underlie the observed hypocholesterolaemia.

In a previous study in which we established blood reference values for healthy boa constrictors, we found evidence that in young boa constrictors, hematopoiesis is more lymphoid-based and becomes more myeloid-based with age, a feature in line with what is known in mammals (e.g., ruminants, dogs) including humans.^46^^,^^47^ To avoid making assumptions about the effects of BIBD itself, we performed multiple regression analysis to assess whether age and sex had any influence on the examined parameters. Indeed, also in the present cohort, independent of BIBD and infection status, the percentage and absolute number of azurophils and heterophils positively correlated with age, whereas the percentage and number of lymphocytes showed a negative correlation (decrease). For AST, the liver enzyme that the older study claimed to be elevated in “acutely infected” snakes,^4^ we observed a significant positive correlation with the male sex, while we could only detect a trend for elevated levels with BIBD. Such a sex-correlated difference is in line with data from humans; the reference values for AST are generally higher for men than for women, apparently for so far unknown reasons.^48^

Another area of scientific interest is the determination and quantification of stress of wild and captive animals. The focus has been on birds and mammals although reptiles have also increasingly gained attention.^49^^,^^50^^,^^51^^,^^52^ However, the definition and “quantification” of stress is challenging in animals, and particularly in reptiles; hence, different approaches have been taken^49^^,^^50^^,^^51^^,^^52^^,^^53^ While some authors focused purely on behavioral changes,^54^ others assessed stress hormone (in particular corticosterone) levels, under baseline and stress-inducing conditions, in plasma and/or feces or combined the latter with the determination of plasma glucose levels and leukocyte counts.^53^^,^^55^^,^^56^ In captive snakes, management related aspects, such as environmental temperature, humidity, enclosure size, and crowding, are described as the main stressors.^57^ In humans, it is widely accepted that chronic or long-term stress can suppress immunity by decreasing immune cell numbers and function and/or increasing active immunosuppressive mechanisms (e.g., regulatory T cells). Chronic stress can also dysregulate immune function by promoting proinflammatory and cytokine-driven responses.^58^ Cytokines do not only regulate cellular interactions within the immune system but also influence the hypothalamic-pituitary-adrenal axis, which is directly associated with the stress response.^59^^,^^60^ Stress may also be relevant in the context of BIBD, as chronically elevated stress levels have been shown to exacerbate immune dysfunction and contribute to the progression of immune-related disorders.^61^ In the present study, we measured three biomarkers formerly suggested as stress markers in wild common garter snakes (*Thamnophis sirtalis*): plasma corticosterone and glucose concentrations, and the whole blood heterophil:lymphocyte ratio.^62^ All three parameters, and in particular corticosterone, showed substantial interindividual variation but did not differ to a statistically significant degree in their mean values between BIBD positive and negative snakes. One reason for the high variation might be the variable circumstances under which the blood samples were taken in the current study. Some animals were sampled at the owners’ premises, where they were handled for a relatively short time (≈15–20 min), while others were sampled directly on site at the university, after being transported for varying periods of time (which could be assumed to subject them to a certain degree of stress). Indeed, serum glucose concentrations in snakes significantly increase 30 min after manipulation,^56^ and corticosterone levels in reptiles can only be considered baseline if blood is collected within 4 min after capture.^63^ Furthermore, due to variable approaches and inconsistencies in the literature, the interpretation of corticosterone levels is not straightforward.^64^ Overall, higher baseline corticosterone concentrations in wild animals are often seen as indicators of increased physiological stress; however, recent studies suggest stress-induced corticosterone may better reflect an organism’s ability to handle stressors.^65^^,^^66^ Plasma corticosterone levels typically rise within 2–5 min after exposure to a stressor and peak at levels 10–100 times higher than baseline within 30–60 min.^67^

The variation in sampling conditions in the present study likely explains the high interindividual variability. Given that this heterogeneity is often inevitable, it would be preferable to use a different type of sample, e.g., feces, in future studies. In fecal samples, hormone levels accumulate over time, whereas plasma concentrations represent a “snapshot”.^55^ In addition, to address the question whether captive *B. constrictor* is indeed chronically stressed, representative data from wild indigenous populations are required. In a study by Sparkman et al. (2014), captivity of wild gravid female snakes *(Tamnophis elegans*) was shown to result in increased plasma corticosterone levels as well as increased heterophil to lymphocyte (H:L) ratios over time.^68^

It is generally accepted that the innate immune response is more relevant/robust in reptiles than its adaptive counterpart.^69^ Still, certain components of the latter (e.g., T helper cells, Ig classes, and MHC class genes) have been described, at genetic and/or functional level.^24^^,^^29^^,^^70^^,^^71^^,^^72^ Therefore, the current study employed functional immune assays that not only target components of the innate (phagocytosis, lysozyme concentration, and hemagglutination/hemolysis) but also the adaptive (leukocyte blast transformation test, immunoglobulin concentrations, and ELISA assay) immune responses. Using an antibody against the monocyte/macrophage marker Iba1 that we employ for immunohistochemistry in tissues and cell pellets,^40^ we established a phagocytosis assay by incubating buffy coat cells with fluorescent beads. This revealed a significant reduction in the number of Iba1-positive cells performing phagocytosis in the peripheral blood of BIBD+ boas. In boa constrictors, Iba1 is expressed by two morphologically—and possibly functionally—very similar but distinct cell types in the blood, namely monocytes and azurophils.^40^ In general, macrophages, monocytes, and heterophils are considered as the major phagocytic cell types in reptiles, with evidence that B cells and thrombocytes also have phagocytic capacity in boas.^73^ Azurophils, however, appear to be a cell type unique to reptiles whose exact origin and function have not yet been elucidated.^74^^,^^75^ Since they express Iba1, we assume that azurophils are (possibly immature) monocytic cells and, based on ultrastructural evidence of phagocytized intracellular material, we concluded that they are capable of phagocytosis.^40^ Considering that monocytes only account for <10% of blood leukocytes, whereas azurophils comprise up to 60%,^40^ the results of the phagocytosis assay, in which an overall high number of phagocytic cells were found to be Iba1-positive, provide strong evidence that azurophils represented the majority of phagocytosing cells in the assay. The overall reduction of phagocytic activity, also in light of the fact that these cells carry viral IB,^25^ is of particular interest considering that infection and inhibition of tissue macrophages (and dendritic cells) is a feature of human arenavirus infections, e.g., with Lassa virus (LASV) and lymphocytic choriomeningitis virus (LCMV). For both these arenaviruses, viral cell entry is mediated by α-dystroglycan, a cellular receptor originating from a highly conserved gene that is expressed on human monocytes/macrophages.^76^ Infection with LASV in humans has been described to reduce the phagocytic activity of dendritic cells and macrophages, which, in the latter, was proposed to be linked to a failure in the maturation process of monocytes.^77^^,^^78^^,^^79^ A more recent study on the modulation of New World arenavirus (e.g., Junín virus, Machupo virus) entry by host proteins further elucidated the mechanism behind the impairment of phagocytosis,^80^ identifying the cell surface attachment factors and receptors that mediate viral cell entry as well as cell-intrinsic molecules, like pattern recognition receptors and restriction factors, that are responsible for “sensing” infection by recognizing viral proteins and nucleic acids as “foreign”. In particular, the authors uncovered two restriction factors that act upon viral entry, tripartite motif protein 2 (TRIM2) and signal regulatory protein alpha (SIRPA). SIRPA is a cell surface protein of macrophages that blocks phagocytosis and works with TRIM2 to decrease arenavirus internalization.^81^ TRIM2 knockout in macrophages also alleviated the SIRPA-mediated inhibition of phagocytosis of apoptotic cells, thus mechanistically linking virus endocytosis and phagocytosis.^80^ The cell entry receptor and/or further co-regulating cell-intrinsic molecules utilized by reptarenaviruses are presently unknown; however, it cannot be excluded that the reduced phagocytic activity of monocytes (and azurophils) in BIBD represents a direct effect of virus infection of these cells.

The present study also revealed significantly lower total immunoglobulin concentrations in the plasma of snakes with BIBD. As shown in *B*. *constrictor* and *C*. *hortulanus*, boid snakes produce IgM, IgD and two subclasses of IgY, IgYa and IgYb.^82^ In an attempt to reveal a potential link between the reduced total immunoglobulin concentration and BIBD itself, we employed our self-made anti-UGV-1 IgY and IgM antibodies to assess the reptarenavirus specific response. This was confirmed in 77% of the snakes with BIBD. Interestingly, anti-UGV-1 IgM antibodies (69%) were detected more frequently than anti-UGV-1 IgY (49%), despite IgY, the functional equivalent of mammalian IgG, being considered the primary defense player in reptiles due to its longer half-life and greater secretion in comparison to IgM.^82^ Virus-specific antibodies were also found in the BIBD−/vRNA− snakes (37%) which suggests infection of these animals at least at an earlier time point. Whether this finding results from viral clearance or from infection with yet undetected reptarenavirus(es) with which the antibodies cross-react but that our multiplex PCR would not pick up would require further investigations.

The virus-specific antibody levels were higher in snakes with BIBD than in those without confirmed reptarenavirus infection, which had the higher overall immunoglobulin concentration in the plasma. As the total immunoglobulin concentration reflects the amount of all antibodies in the plasma, pathogen-specific and not, multiple factors are likely to contribute to the observed result. One possibility is that reptarenavirus infection would dampen the antibody production in general. While there is evidence that certain Old World arenaviruses, such as LASV, have evolved mechanisms to suppress or evade the production of neutralizing antibodies targeting the viral glycoprotein,^83^ no studies have yet examined the overall efficiency of the humoral immune response during chronic arenavirus infection. Another option is that circulating immune complexes, formed of reptarenavirus specific antibodies with virions and/or viral antigens, may account for a reduction of total immunoglobulins. Studies on persistent LCMV infections in mice have shown that antibodies produced against the virus form immune complexes, which are then deposited in the renal glomeruli. This deposition reduces the levels of circulating antibodies.^84^^,^^85^^,^^86^ Although, we cannot exclude the possibility that antigen-antibody complexes may interfere with the measurement of total immunoglobulin levels in our study, we consider this unlikely, as similar trends would have been expected in the specific antibody measurements. To better understand these dynamics, future more detailed analyses of individual immunoglobulin fractions are crucial.

Lastly, the present study also shows that the *B. constrictor* possesses functional natural antibodies (NAbs) that mediate complement activation during the HA/HL test performed with sheep blood. Interestingly, NAbs, most of which are pentameric IgM, were suggested to compensate for the decreasing sensitivity of the adaptive immune system observed in aging pythons.^87^ NAbs also seem to play a protective role during LCMV infections in mice by mediating epitope recognition.^88^ Further studies are needed to elucidate if NAbs play a comparable role in reptarenavirus infection and development of BIBD in snakes.

In addition, it is worth noting that, aside from a higher lysozyme activity observed in female *B. constrictor*, immune function assays in the studied cohort were not significantly influenced by sex or age. Previous studies examining the effects of aging on immune function in reptiles have produced mixed results: while some report no age-related differences in innate immune responses,^89^^,^^90^ others describe a decline, particularly in females, where immune function is suspected to be compromised due to the increase in reproductive effort with advancing age.^89^^,^^90^^,^^91^ In a recent study, we found evidence that the lymphoid organs of *B. constrictor*, including the spleen and thymus, undergo age-related morphological changes, represented by a decrease in cellularity and increased connective tissue deposition.^92^ Interestingly, despite these structural alterations, the lymphoid organs of this species appear to maintain their immunological competence, which is especially notable given the apparent lifelong reproductive activity reported by breeders (personal communication). This is also an interesting area for future studies.

In conclusion, our study provides evidence that BIBD in *B. constrictor* is indeed associated with alterations in immune function, particularly in the phagocytic activity of the monocytes and macrophages. The lower percentages of Iba1-positive phagocytic cells and circulating monocytes suggest that BIBD impairs key aspects of the innate immune response, potentially explaining the apparent increased susceptibility to bacterial infections in boas with BIBD. Future studies should investigate the temporal relationship between impairment of phagocytic activity and disease progression, to determine whether reduced phagocytosis precedes overt clinical disease and could serve as an early indicator of BIBD progression. While experimental infection studies would provide a valuable approach to further investigate the role of secondary infections in disease development, their implementation is not without challenges. Previous experimental infection studies have highlighted several caveats, including limited reproducibility and considerable variability in infection and disease outcomes depending on factors such as host species, viral isolate, and inoculation route.^7^^,^^14^

Furthermore, experimentally inducing secondary infections requires prior knowledge of which pathogens are most likely to contribute to or trigger clinical disease, which to date also remains poorly understood. In addition, given the metabolic alterations observed in BIBD, such as hypocholesterolaemia, it could be of interest to investigate whether and in what way reptarenavirus infection alters cellular function. In future studies, for example, investigations could focus on whether the co-localization of the viral nucleoprotein with mitochondria leads to mitochondrial dysfunction and whether this, in turn, contributes to alterations in cellular cholesterol metabolism. Together, these approaches would further enhance our understanding of BIBD pathogenesis and could ultimately help to develop strategies to manage the disease in captive populations.

### Limitations of the study

The current study was undertaken on naturally infected animals that were euthanized after the diagnosis of BIBD. It therefore lacks a temporal dimension, both on the length of infection and on the potential clinical course after occurrence of IBs. Prospective longitudinal studies would be necessary to determine whether immunological alterations, such as reduced phagocytic activity, develop prior to the presence of IB and could potentially align with disease progression.

The number of reptarenavirus-positive snakes without IB, i.e., BIBD-negative, was very small which limits meaningful statistical comparisons. In our experience, such infected but not (yet) diseased animals are only detected when larger groups of boas, i.e., in breeding colonies, are screened for BIBD. Such screening attempts are not common and the logistics to keep these animals, optimally in quarantine, and repeatedly subject them to blood examinations until the time when they might eventually develop BIBD are extremely challenging. However, such carrier animals are of particular interest for understanding disease pathogenesis, and we are keen to investigate these to also generate a pool of data that would allow firm conclusions regarding the functional differences between infection with and without disease.

We also performed an exploratory comparison between the five BIBD-positive animals that had shown clinical signs and the clinically unaffected BIBD-positive animals. This did not indicate significant differences in any of the examined hematological or biochemical parameters. However, the very small number of clinically affected animals substantially limits the statistical power of this comparison, hence no firm conclusions can be drawn regarding potential associations between clinical disease and the measured parameters.

## Resource availability

### Lead contact

Requests for further information and resources should be directed to and will be fulfilled by the lead contact, Eva Dervas (eva.dervas@uzh.ch).

### Materials availability

This study did not generate new unique reagents.

### Data and code availability

• All data supporting the findings of this study are available within the article and its supplemental information files and from the corresponding author upon reasonable request.
• This paper does not report original code.
• Any additional information required to reanalyze the data reported in this paper is available from the lead contact upon reasonable request.

## Acknowledgments

We are grateful to the technical staff of the Histology Laboratory, Institute of Veterinary Pathology, Vetsuisse Faculty, University of Zurich, Switzerland, and Katja Pohle from the Leibniz Institute for Zoo and Wildlife Research, Germany, for excellent technical support. We would also like to thank the breeders who provided the animals for this study.

## Author contributions

Conceptualization, E.D. and A.K.; methodology, G.A.C., E.D., E.M., A.L., M.N., F.B., U.H., J.H., and F.S.; investigation, E.D. and F.B.; data curation, E.D., G.A.C., and F.B.; writing – original draft preparation, E.D.; writing – review and editing, A.K., G.A.C., and J.H., E.D., M.N., A.L., F.B., and U.H.; visualization, E.D. and U.H.; project administration, A.K. All authors have read and agreed to the published version of the manuscript.

## Declaration of interests

The authors declare no competing interests.

## STAR★Methods

### Key resources table

**Table:** 

REAGENT or RESOURCE	SOURCE	IDENTIFIER
**Antibodies**		

Anti-Iba1 antibody, rabbit polyclonal	FUJIFILM Wako Pure Chemical Corporation	Cat# 019-19741; RRID: AB_83950
Donkey anti-goat IgG, Alexa Fluor 488	Thermo Fisher Scientific/Invitrogen	Cat# A-11055; RRID: AB_2534102
Anti-boa IgM antibody	Korzyukov et al.^70^	N/A
Anti-boa IgY antibody	Korzyukov et al.^70^	N/A

**Bacterial and virus strains**		

UGV-1 virions	This study	Windbichler et al.^24^
Micrococcus lysodeikticus ATCC 4698, lyophilized cells	Sigma-Aldrich	Cat# M0508-1G

**Biological samples**		

Boa constrictor blood samples	This study	128 animals; see Table S1
Sheep red blood cells	Innovative Research, Inc.	SKU: ISHRBC100P15ML

**Chemicals, peptides, and recombinant proteins**		

RPMI-1640 medium	Sigma-Aldrich	Cat# R8758
Fetal bovine serum (FBS)	Sigma-Aldrich	Cat# F7524
Penicillin/streptomycin/glutamine	Bioswisstec	Cat# EFA 2213B
Phytohemagglutinin-M (PHA-M)	Sigma-Aldrich	Cat# L8902
Hen egg white lysozyme	Sigma-Aldrich	Cat# L6876
Drabkin’s solution	Sigma-Aldrich	Cat# D5941
Natt and Herrick solution	Bioanalytic GmbH	Cat# 004025–0100
Modified Wright-Giemsa stain/Hematek Stain Pak	Siemens Healthcare	Cat# 158058
Fluoresbrite YG Microspheres, 1.00 μm	Polysciences, Inc.	Cat# 17154
DAPI	Novus Biologicals	Cat# NBP2-31156
Paraformaldehyde (PFA), 95% powder	Sigma-Aldrich	Cat# 158127
1-Step TMB ELISA Substrate Solution	Thermo Scientific	Cat# 34028
Bovine serum albumin (BSA)	Merck	Cat# A7030

**Software and algorithms**		

ImageJ	National Institutes of Health	Version 1.48
Stata Statistical Software	StataCorp	Version 13
R	R Foundation for Statistical Computing	Version 2024
RStudio	Posit Software, PBC	Version 2024.09.1
changepoint package	CRAN	Version 2.3

### Experimental model and study participant details

#### Study animals and ethical approval

The study was undertaken on blood samples of 128 captive *B. constrictor* from 12 private collections and breeding colonies in Switzerland and Germany (Table S1), and a reptile shelter (A) in Switzerland that takes over reptiles from private owners or breeders who are unable to maintain their collections. The latter snakes were euthanized as the shelter had not succeeded in rehoming the animals over a prolonged period of time. They were subjected to a complete postmortem examination, including blood sampling by cardiocentesis, to gain information on the general disease status in the shelter (presence of BIBD/reptarenavirus infection or other viral/bacterial diseases of which the shelter owners might not have been aware). For these animals, information on their exact origin was not available anymore.

The animals from the German breeding colony (B) had previously been confirmed to be BIBD positive, based on the presence of the characteristic intracytoplasmic IB in blood cells in a peripheral blood smear that was examined as part of a diagnostic screening of the entire colony (data also published in^39^). As the owners wanted to clear the collection from BIBD, they submitted the BIBD positive snakes for euthanasia and postmortem examination, to confirm the disease and to determine any other (associated/secondary/concurrent) pathological conditions.

The remaining animals, from Swiss breeders/private owners (C-L), were either blood sampled as part of a BIBD screening scheme for the collection or were submitted by the owners for euthanasia and subsequent diagnostic postmortem examination.

Animals were maintained under husbandry conditions that varied among the respective private collections, breeding colonies, and the shelter. Detailed information on the individual husbandry conditions was not available for all animals.

Blood sampling, as part of the BIBD screening scheme, was undertaken under the project licenses ZH 195/2016 and ZH136/2020 from the Cantonal Veterinary Office in Zurich. All snakes submitted alive for postmortem examination were euthanized by veterinarians according to the ASPA Animals Scientific Procedures Act) 1986, Schedule 1 (appropriate methods of humane killing, https://www.legislation.gov.uk/ukpga/1986/14/schedule/1) procedure, and necropsied upon the owners’ request. All diagnostic blood samples were examined with approval by the institutional ethics review board (MeF-Ethik-2024–01). Also, the terms of service to which owners agree when submitting an animal for a diagnostic postmortem examination include the permission to make use of material from the examination for both teaching and research.

A subset of the animals included in this study was also part of two previously published investigations: one focusing on reference values of blood parameters in clinically healthy, BIBD- and reptarenavirus-negative *B. constrictor*, and the other examining hemolymphatic tissues in both BIBD- and reptarenavirus-negative, and BIBD-positive individuals.^25^^,^^40^ Table S1 provides animal data, including sex and age, clinical signs, examinations and a reference linking the animal numbers used in this study to those in the earlier publications. Animal age and sex were taken into consideration in the statistical analyses where applicable.

### Method details

#### Blood sampling, euthanasia and postmortem examination

As part of the BIBD screening scheme, the blood was collected from the animals on the owners’ premises, to keep manipulation/transport, and therefore stress, at a minimum, unless the owner preferred the alternative option, i.e., blood sampling at the Institute of Veterinary Pathology Zurich. Blood was collected by cardiocentesis using a 22- or 25-gauge needle on a 3 mL syringe. The heart was visualized by a SonoTrax Vascular Doppler (FS15575, EDAN, USA). A blood volume of 1–2 mL was collected into heparinized tubes (Sarstedt, Germany), the volume depending on the size of each animal but not exceeding 8% of the total blood volume.^93^ When animals were submitted for euthanasia and a full postmortem examination for diagnostic purposes, the animals were euthanized according to the ASPA, Animals (Scientific Procedures) Act 1986, schedule 1 (appropriate methods of humane killing, https://www.legislation.gov.uk/ukpga/1986/14/schedule/1) procedure, following previously published protocols.^25^ In the first 1–2 min after decapitation, blood (0.5–3 mL) was collected by cardiocentesis using a 22- or 25-gauge needle on a 3 mL syringe.

#### Processing of blood samples

From all blood samples, four smears were prepared and stained within 6 h of sampling with an automated slide stainer (HEMA-TEK 2000 slide stainer, Bayer HealthCare AG, Berlin, Germany), using a modified Wright-Giemsa staining kit (Hematek® Stain Pak, Siemens Healthcare Inc., New York, USA), and air dried. The stained smears were subjected to a cytological examination to determine the BIBD status of the animals (see Diagnosis of reptarenavirus infection and BIBD) and to perform a differential leukocyte count (see Basic haematological parameters). Additionally, within 6 h of sampling, 2 mL aliquots of blood were collected into heparinized tubes (Sarstedt) and centrifuged in a microcentrifuge (LLG-uniCFUGE 2; Faust, Schaffhausen, Switzerland) at 1,200 g for 5 min at room temperature (RT), for complete separation of red blood cells and plasma. One aliquot was subsequently processed for the phagocytosis assay (see Phagocytosis assay) while the plasma was collected and stored at −80 °C in 200 μL aliquots for further analysis.

#### Diagnosis of reptarenavirus infection and BIBD

All animals in this study were tested for reptarenavirus infection by a multiplex RT-PCR assay that detects all currently known reptarenaviruses, performed on the whole blood, following a recently published protocol.^38^ For the diagnosis of BIBD, a blood smear stained with the Modified Wrights's stain (Siemens Healthineers, Erlangen, Germany) was screened for the presence of IB in blood cells (erythrocytes and white blood cells); this approach is reliable and considered the “gold standard” for diagnosing the disease.^12^^,^^24^^,^^39^

#### Blood parameters

##### Basic haematological parameters

In 88 individuals (BIBD+: *n* = 38; BIBD-/reptarenavirus (v)RNA-: *n* = 47; BIBD-/vRNA+: *n* = 3) a range of haematological parameters were assessed (Table S1). Total leukocyte counts as well as heterophil, azurophil, lymphocyte, eosinophil, basophil and monocyte counts were obtained by a 100 white blood cell differential count performed twice on a May Grunwald Giemsa stained blood smear (HEMA-TEK 2000; Siemens Healthcare GmbH, Erlangen, Germany). The mean of the two differential counts was calculated to obtain a percentage for each cell type. The Packed cell volume (PCV) was determined by placing whole blood into a plain glass microhaematocrit tube (Arnold Bott AG, Glattbrugg, Switzerland) and spinning at 12,000 g for 5 min using a microhaematocrit centrifuge (Haematocrit 210 Centrifuge, Hettich, Bäch, Switzerland). Total white blood cell and red blood cell counts were obtained using a 1:200 blood dilution with Natt and Herrick solution and an improved Neubauer haemocytometer, as previously described.^94^ Haematocrit, hemoglobin and mean cell haemoglobin concentration (MCHC). The hemoglobin concentration was assessed with the cyanmethaemoglobin method and measured using a photometer (Photometer LP 400; Dr. Lange, Burladingen, Germany). Briefly, after lysis of the red blood cells using Drabkin’s solution followed by centrifugation of the lysate at 12,000 g for 5 min, a colourimetric measurement at 540 nm was performed. The MCHC was calculated from the PCV and the hemoglobin concentration. The absolute number of each white blood cell type was calculated from the total leukocyte counts and the respective relative numbers.

#### Clinical chemistry

In 114 animals ((BIBD+: *n* = 43; BIBD-/vRNA-: *n* = 69; BIBD-/vRNA+: *n* = 2, Table S1), the following biochemical parameters were measured: total protein (TP), cholesterol, aspartate transaminase (AST), albumin (ALB), lactate dehydrogenase (LDH), uric acid and glucose. TP, cholesterol, AST, LDH and uric acid. All parameters were determined by colourimetry with an autoanalyser (Cobas Mira Roche autoanalyser, Hoffmann-La Roche Ltd, Basel, Switzerland), using commercially available kits (AST, cholesterol, LDH, TP, ALB: Diatools AG, Villmergen, Switzerland; uric acid: AxonLab, Dättwil, Switzerland).

#### Assessment of stress parameters

For a better understanding of both acute and chronic stress, we examined the corticosterone and glucose concentration in the plasma as well as the heterophil/lymphocyte (H/L) ratio, an approach similar to a previous study performed in the common garter snake (*Thamnopsis sirtalis*).^62^ Together, these markers may provide a comprehensive picture of the stress response, as they represent aspects of the physiological stress axis (corticosterone and glucose) and the immune response (via H/L ratio). Corticosterone levels were determined in the plasma of 67 animals (BIBD+: *n* = 29; BIBD-/vRNA-: *n* = 35; BIBD-/vRNA+: *n* = 3, Table S1). Plasma samples were analyzed using a previously described enzyme-immunoassay.^95^ An aliquot of 150 μL of plasma was admixed with 3 mL of diethyl ether, and the samples were analyzed in duplicates. Serial dilutions of serum samples yielded profiles that paralleled the standard curve.

Glucose levels were measured using a Roche Cobas c501 analyser (Roche Diagnostics AG, Rotkreuz, Switzerland). The heterophil/lymphocyte ratio was calculated based on the haematological parameters (see Basic haematological parameters).

#### Evaluation of the immune response

Given the limited understanding of the specific immune components targeted by reptarenavirus infection, we aimed to assess a broad range of immune functions, adressing both the innate and adaptive immune response. To ensure the tests would generate reliable data, we mainly selected methods that had been previously established for use in reptiles and, where available, specifically in snakes.

#### Phagocytosis assay

Using a previously published method for the assessment of phagocytosis in reptilian B cells^96^ as the basis, we developed a protocol to quantify the *in vitro* phagocytic ability of Iba1-positive cells isolated from the peripheral blood (representing monocytes and azurophils, as shown in^40^). This was performed on samples from 82 individuals (BIBD+: *n* = 33; BIBD-/vRNA-: *n* = 48; BIBD-/vRNA+: *n* = 1; Table S1). An aliquot of heparinized, centrifuged blood (4,000 g for 5 min; Micro Centrifuge, LabLogisticsGroup, Meckenheim, Germany) was utilized for the isolation of blood leukocytes (as described under 2.3). Twenty μL of the resulting buffy coat (visible as a thin layer above the red blood cells) were pipetted off and resuspended in 500 μL RPMI cell culture medium (RPMI 1640 medium, Gibco, Schwerte, Germany) supplemented with 5% fetal bovine serum (FCS) and 1% penicillin (Gibco)/streptomycin (Gibco)/glutamine (Bioswisstec, Schaffhausen, Switzerland). The cell suspension was then transferred to a 24-well plate (Techno Plastic Products, Houston, Texas, USA; 2 wells per sample; duplicates) after a glass coverslip was placed in each well. Each cell suspension was then supplemented with 40 μL of fluorescent beads (1 μm diameter; 6.4 × 10^7^ beads/mL; Fluoresbrite Plain Yellow Green Microspheres, Polysciences, Hirschberg, Germany) and incubated for 3 h at 27 °C. After incubation, the supernatant was removed from each well, and the wells were washed twice with PBS. For permeabilization of the cell membranes, the cells were incubated with 200 μL of 0.3% Triton-X (Sigma-Aldrich) for 7 min at RT, then washed twice with PBS and fixed for 15 min with 4% paraformaldehyde (Sigma-Aldrich), pH 7.4 at RT. Coverslips were removed from the 24-well plates, incubated overnight at 4 °C with goat anti-Iba1 antibody (Wako; 1:400 in PBS), then washed five times with PBS, and incubated for 30 min at RT with Alexa Fluor 488-labeled donkey anti-goat immunoglobulin (Invitrogen, Waltham, USA; 1:500 in PBS), followed by a 15 min incubation with DAPI (4 = ,6-diamidino-2-phenylindole; Novus Biologicals, Centennial, USA; 1:10,000 in PBS). The coverslips were washed twice with distilled water and placed on a glass slide with FluoreGuard mounting medium (Biosystems, Muttenz, Switzerland). Slides were examined with a Nikon Eclipse Ni-U microscope with NIS Advanced Research software at a wavelength of 460 nm. The number of Iba1-positive cells containing fluorescent beads and the total number of cells containing fluorescent beads were counted. The percentage of fluorescent bead-containing cells that were Iba1-positive was calculated by dividing the number of Iba1-positive cells containing fluorescent beads by the total number of cells containing fluorescent beads, multiplied by 100.

#### Leukocyte blast transformation test

The leukocyte blast transformation test measures the *in vitro* reactivity of mononuclear cells to sensitizing (*in vivo* encountered) antigens or mitogens.^97^ The method is based on the quantification of cell growth by measuring glucose consumption.^49^ The assay was applied to samples from 46 animals (BIBD+: *n* = 13; BIBD-/vRNA-: *n* = 30; BIBD-/vRNA+: *n* = 3; Table S1). A fresh (within 6 h after collection) whole blood sample (120–280 μL, depending on the amount of blood available) was diluted 1:5 in RPMI 1640 (Sigma-Aldrich), supplemented with 5% FCS and antibiotics (1000 IU penicillin and 1000 μg streptomycin/mL). Of this, 40 μL were frozen immediately at −80 °C for determination of the initial glucose concentration. The remaining total volume was then divided in order to establish two conditions: 1) untreated control cultures (duplicates or triplicates), and 2) treated cultures (duplicates or triplicates), by pipetting 200 μL per well to 2–6 wells of a sterile 96-well plate (Thermo Scientific, Waltham, MA, USA). The (stimulated) cultures were supplemented with phytohaemagglutinin-M (PHA; Sigma-Aldrich; 1 μL per well) and the plates incubated at 30 °C in a 5% CO_2_ atmosphere for 48 h.

The glucose concentration was measured in the initial medium and in the treated and untreated control samples after 48 h, using a standard (100 mg/dL) glucose solution, by means of an ortotoluidine colouanalysrimetric test. For this, 12.5 μL of the culture supernatant was transferred to 0.5 mL of ortotoluidine reagent (Sigma-Aldrich), boiled for 8 min, cooled rapidly in cold water, and read in a spectrophotometer at 610 nm wavelength (Synergy HT, BioTek Instruments GmbH, Basel, Switzerland), using the reagent as a blank. The transformation index (IS) was calculated as follows: T I % = [(RG−S G)/RG]×100, with TI: blast transformation index, RG: glucose concentration in the initial RPMI 1640 culture medium, and SG: glucose concentration in the control (M) or phytohaemagglutinin-(PHA-) treated sample after incubation.

#### Total immunoglobulin measurement

The total immunoglobulin concentration was measured in the plasma of 91 snakes (BIBD+: *n* = 30; BIBD-/vRNA-: *n* = 56; BIBD-/vRNA+: *n* = 1; Table S1) using a published method applied to the blood of viperid snakes,^97^ based on the fact that at pH 7.4, metal salts with concentrations as low as 24 mg/L precipitate immunoglobulins.^98^ To achieve precipitation, a 0.024% (w/v) barbital buffer zinc sulfate solution was prepared. For this, one liter of deionized water was boiled for 10–15 min and 208 g of zinc sulfate were added. The solution was left to incubate at RT for 30 min. Afterward, 6.6 μL of plasma was mixed with 193.4 μL of the barbital buffer zinc sulfate solution and allowed to precipitate for 30 min at RT. The optical density was measured in a spectrophotometer at 475 nm wavelength (Synergy HT).

#### Lysozyme assay

Lysozymes are bactericidal enzymes contributing to the innate immunity that are ubiquitous in animals, plants, and microorganisms.^99^ Their bactericidal activity relies on degradation of the bacterial cell wall, a process catalyzed by hydrolysis of the peptidoglycan layer, the main component of the cell wall of Gram-positive bacteria, but also present in lower amounts in Gram-negative bacteria.^99^ The lysozyme concentration in the blood reflects the level of innate response activation, particularly the presence of inflammation or infection.^100^ The lysozyme concentration in the plasma was determined in 79 individuals (BIBD+: *n* = 35; BIBD-/vRNA-: *n* = 43; BIBD-/vRNA+: *n* = 1; Table S1). The lysoplate assay method was applied.^101^^,^^102^ For this assay, 30 mL of 1% Noble agar gel (Sigma-Aldrich) containing 0.5 mg/mL lyophilized *Micrococcus lysodeikticus* (Sigma-Aldrich) was pipetted into sterile 150 × 20 mm Petri dishes (Sarstedt, Nümbrecht, Germany). Test holes were punched into the gel with a pipette tip. After solidification, 10 μL of plasma was pipetted into the test holes on the plates. Crystalline hen egg white lysozyme (Sigma-Aldrich) was used at a concentration of 0.5, 0.8, 1, 2, 4, 8, 10, 20, 40 and 100 μg/mL to prepare a standard curve for each plate. Plates were incubated at 30 °C for 20 h. During incubation, a clear zone developed in the area of the gel surrounding the sample inoculation site, corresponding to bacterial lysis. The diameters of the cleared zones are proportional to the log of the lysozyme concentration.^102^ Plates were photographed in a photobox (Imaging system; peqlab, Erlangen, Germany) with a ruler next to them as a reference scale. The diameter of the lytic areas was measured digitally using the software ImageJ (version 1.48) and the obtained values converted into hen egg lysozyme equivalents (HEL equivalents, expressed in μg/mL) according to the standard curve.^103^

#### Haemolysis-haemagglutination assay

Natural antibodies (Nab), measured as haemagglutination (HA) titers, bind non-specifically to various antigens and have important roles in opsonization. Haemolysis (HL), mediated by complement, is part of the innate immune system, and its activation results in lysis of opsonized pathogens. The Nab and complement titers were measured in 78 individuals (BIBD+: *n* = 35; BIBD-/vRNA-: *n* = 43; BIBD-/vRNA+: *n* = 1; Table S1), using a modified haemolysis-haemagglutination assay^103^ that had previously been adapted to various reptile species.^91^^,^^104^^,^^105^ After pipetting 20 μL of plasma into the first two rows of a U-shaped 96-well microtitre plate (Sigma-Aldrich), 20 μL sterile PBS were added to columns 2–12. The content of the second row of wells was serially diluted (1:2) with PBS until the 11^th^ column, resulting in a dilution series for each sample from 1/2 to 1/1024. The last column of the plate was used as a negative control, containing only PBS. Subsequently, 20 μL of 1% sheep red blood cell suspension (Innovative Research; Peary court Novi, USA) were added to all wells and the plates were incubated at 30 °C for 90 min. Afterward, in order to increase the visualization of agglutination, the plates were tilted at a 45-degree angle and incubated in this position at RT. After 20 min incubation, the agglutination activity was determined, followed by evaluation of the lysis activity after 90 min incubation. The Nab and complement titers were presented as the log_2_ of the reciprocal of the highest dilution of plasma showing positive haemagglutination or lysis, respectively.^103^

#### Enzyme-linked immunosorbent assay (ELISA) for anti-UGV-1 Ig M and IgY antibodies

To assess the antibody response of the snakes of this study against the virus, we employed custom-made antibodies targeting S- and L segments of reptarenaviruses (in particular the University of Giessen virus-1, UGV-1), as previously described.^106^ The anti-UGV-1-IgM and IgY levels were measured in the plasma of 99 individuals (BIBD+: *n* = 36; BIBD-/vRNA-: *n* = 60; BIBD-/vRNA+: *n* = 1; Table S1) with a previously established ELISA using concentrated UGV-1 virions as antigen.^24^^,^^107^ Briefly, UGV-1 virions were diluted 1:400 in 0.05 M carbonate buffer, pH 9.6, and used to coat Immuno Breakable Modules Clear Lockwell C8 Polysorp plates (Thermo Scientific), followed by overnight incubation at 4°C. After coating, 1% (w/v) bovine serum albumin (BSA) in PBS solution was applied for blocking (2 h at 37°C), followed by washing with Tris-buffered saline (TBS) + 0.05% Tween 20 (TBS-T)) and incubation with the plasma samples (diluted 1:400) in 0.25% BSA/PBS (1 h at 37°C). After four TBS-T washes the plates were incubated for 45 min at 37°C with horseradish peroxidase (HRP) labeled anti-boa IgM or anti-boa IgY antibodies (described in,^70^ diluted 1:2000 in 0.25% BSA/PBS), washed four times with TBS-T and incubated with 100 μL/well 1-Step TMB ELISA Substrate Solution (Thermo Scientific) for 20 min at RT. The reaction was terminated by addition of 50 μL/well 0.5 M H_2_SO_4_, and the results were read (OD at 450 nm) with a Multi-Mode Microplate Reader (BioTek Synergy HT; Winooski, Vermont, USA).

For determination of positive and negative ELISA results, a change point analysis utilising the changepoint v.2.3 package^108^ was performed with R,^109^ using RStudio (Integrated Development Environment for R, version 2024.09.1 Build 394, Posit Software, PBC, Boston, Massachusetts, USA) to set the separate cut-off values for IgM and IgY, as previously described.^70^ Briefly, the cpt.meanvar function was used with the AMOC method on the ELISA data arranged in ascending order. The change points resulted in the OD value of 0.086 for IgM and 0.2218 for IgY. The cut-offs were set just above the detected change point, so that the value at change point was considered negative.

### Quantification and statistical analysis

All haematological, plasma biochemical, stress, morphometric and immune function parameters were analyzed using Stata13 (StataCorp. 2013. Stata Statistical Software: Release 13. College Station, TX: StataCorp LP.). The level of significance testing was set with a *p* value of 0.05. Statistical significance is indicated by asterisks as follows: ∗*p* < 0.05, ∗∗*p* < 0.01, and ∗∗∗*p* < 0.001. Descriptive statistics were applied, and the data were tested for normality by the D'Agostino-Pearson normality test. Normally distributed data were analyzed using a two-sample *t* test. Non-parametric tests (Wilcoxon rank-sum/Mann-Whitney) were used for data that were not normally distributed. Geometric and arithmetic means, including confidence intervals, were determined for logarithmically transformed and raw data, respectively. Medians were reported where non-parametric tests were used.

Multiple linear regression was used to test if reptarenavirus infection and BIBD status, age and sex significantly predicted changes in the haematological, plasma biochemical, stress and immune function parameters. For the multiple linear regression age was transformed (square root) while in other analyses it was divided in two age groups, young animals (<3 years of age; prior to definite sexual maturity and reproduction) and adult animals (≥3 years of age; sexually mature, reproducing animals), as applied in former studies.^25^^,^^40^ Linearity was confirmed using the qnorm plot and the Shapiro-Wilk W test on residuals. The standard error was adjusted for clustering according to the number of breeders involved.

Normally distributed variables include the number of leukocytes, the percentage of azurophils and lymphocytes, haematocrit, hemoglobin, MCHC, total protein, albumin, glucose, heterophil/lymphocyte ratio, the percentage of phagocytic Iba1 positive cells and the Blast transformation index of LBTT (control and PHA-stimulated). Logarithmic transformation was applied to the data for the number of heterophils, eosinophils, basophils, azurophils and lymphocytes, the percentage of heterophils, eosinophils and monocytes, cholesterol, urea, AST, LDH, uric acid, corticosterone, lysozyme and total immunoglobulins. Nonparametric tests were used for the percentage of basophils, the number of monocytes and the HA/HL.

The results are based on statistical analyses between two groups, either BIBD-negative (BIBD-/vRNA-) or positive (BIBD+) snakes, or reptarenavirus-positive and -negative snakes, and significant differences, if present, are commented on in that order. The summary of all results obtained from the statistical analyses is provided in Tables S2–S7. The different group comparisons were undertaken because for each comparison the BIBD negative, reptarenavirus positive animals (BIBD-/vRNA+) were first assigned to the BIBD negative group and then to the reptarenavirus positive group, as, due to the very small sample size (*n* = 3), they could not be considered as a separate group for statistical analysis (low statistical power). However, as carrier animals are of great interest for disease development, given that they might represent an early stage of disease, they are shown as a separate group in some graphs (boxplots), to illustrate their status in relation to BIBD+ and BIBD-/vRNA-animals for certain parameters. To assess whether BIBD+ snakes with clinical signs differed from BIBD+ snakes without clinical signs and from BIBD-negative (BIBD-/vRNA-) snakes, subgroup analyses were performed. For each parameter, comparisons among the three groups were conducted using the Kruskal–Wallis test followed by Dunn’s multiple comparisons test. Adjusted *p*-values from Dunn’s test are reported in Table S7. Given the limited sample size of BIBD+ snakes with clinical signs (*n* = 5) and the number of parameters examined, these subgroup analyses were considered exploratory and interpreted with caution.

## Supplemental information

Document S1. Figure S1 and Tables S2–S7

Table S1. Case material of the study, examinations performed, reptarenavirus infection and BIBD status, results of the ELISA (anti-UGV-1-Ig) and corresponding individual case numbers for the animals in two prior studies by Dervas et al.^33^ and Dervas et al.^20^
