# Supplementary material for: Boid inclusion body disease in Boa constrictor: Unraveling the myth of immunosuppression
Source: iScience. 2026 Sep 22;29(10):117669. doi: 10.1016/j.isci.2026.117669 (PMC13657219; doi:10.1016/j.isci.2026.117669)
Supplement: Supplementary file 1 [file mmc1.pdf]

iScience, Volume *29*

Supplemental information

Boid inclusion body disease in *Boa constrictor*: Unraveling the myth of immunosuppression

Eva Dervas, Gábor Árpád Czirják, Eleni Michalopoulou, Annette Liesegang, Marilisa Novacco, Franz Schwarzenberger, Udo Hetzel, Jussi Hepojoki, Francesca Baggio, and Anja Kipar

Supplemental Material

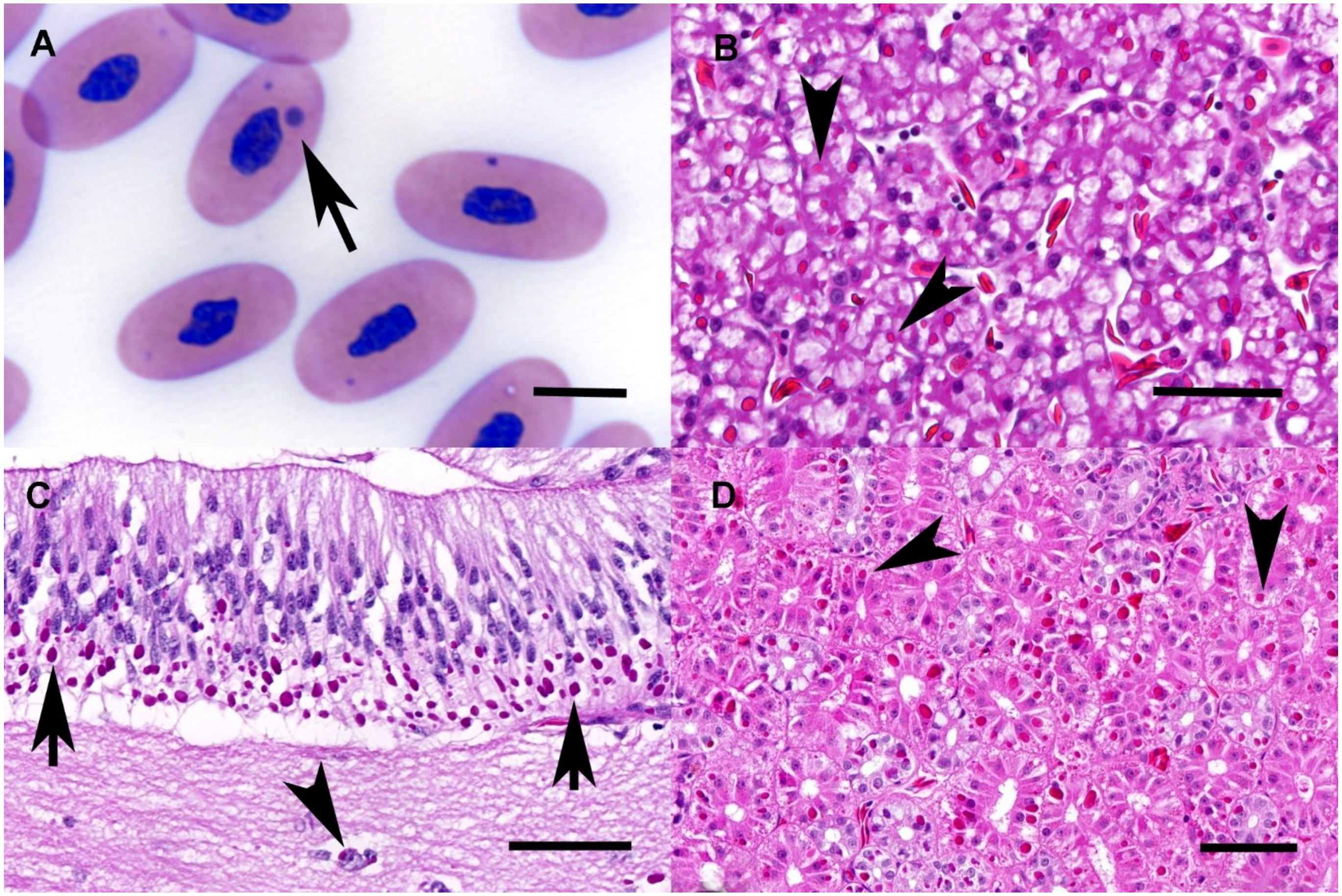

Figure S1. Intracytoplasmic inclusion bodies (IB) in snakes with BIBD. A. Peripheral blood smear (animal B13; 2 years, male). IB in the cytoplasm of an erythrocyte (arrow), Modified Wright's stain. **B.** Liver (animal B6; 2 years, female). Numerous IB in hepatocytes (arrowheads). HE stain. **C.** Brain (animal B4; 2 years, female). Numerous IB in ependymal cells (short arrows) and neurons (arrows). HE stain. **D.** Kidney (animal B13; 2 years, male). Numerous IB in tubular epithelial cells (arrowheads). HE stain. A: scale bar = 10 µm; B-D: scale bars = 50 µm.

	BIBD-<br>(Mean, n, 95%CI)	BIBD+<br>(Mean, n, 95%CI)	vRNA-<br>(Mean, n, 95%CI)	vRNA+<br>(Mean, n, 95%CI)	Total<br>(Mean, n, 95%CI)
Leukocytes $10^3/\mu l$ (t, df, p)	8768.378 (47)<br>(7254.04 – 10596.43)	10600.57 (41)<br>(8521.536 – 13186.85)	8.951 (44)<br>(7364.412 - 10881.08)	10248 (44)<br>(8288.855 – 12672.12)	9578.3 (88)<br>(8310.255 – 11039.83)
	t=-1.3315, df=86, p=0.1865		t=-0.9464, df=86, p=0.3466		
Heterophils $10^3/\mu l$* (t, df, p)	1616.28 (46)<br>(1324.722 – 1972.007)	1448.127 (40)<br>(1217.126 – 1722.971)	1656.9 (43)<br>(1343.048 – 2044.094)	1423.495 (43)<br>(1208.299 – 1677.292)	1535.769 (86)<br>(1346.559 – 1751.566)
	t=0.8271, df=84, p=0.4105		t=1.1502, df=84, p=0.2533		
Heterophils (%) * (t, df, p)	18.184 (47)<br>(15.343 – 21.550)	13.918 (41)<br>(10.924 – 17.732)	18.2388 (44)<br>(15.2651 – 21.792)	14.1306 (44)<br>(11.242 – 17.763)	16.054 (88)<br>(13.893 – 18.551)
	t=1.8591, df=86, p=0.0664		t=1.7755, df=86, p=0.0794		
Eosinophils $10^3/\mu l$* (t, df, p)	172.58 (32)<br>(115.413 – 258.046)	166.991 (30)<br>(126.749 – 220.010)	177.671 (31)<br>(117.733 – 268.123)	162.373 (31)<br>(123.656 – 213.212)	169.850 (62)<br>(133.624 – 215.896)
	t=0.1359, df=60, p=0.8924		t=0.3726, df=60, p=0.7108		
Eosinophils (%) * (t, df, p)	1.895 (32)<br>(1.365 – 2.632)	1.480 (30)<br>(1.185 – 1.849)	1.979 (31)<br>(1.427 – 2.744)	1.429 (31)<br>(1.139 – 1.793)	1.682 (62)<br>(1.380 – 2.049)
	t=1.2566, df=60, p=0.2138		t=1.6698, df=60, p=0.1002		
Basophils $10^3/\mu l$* (t, df, p)	199.782 (42)<br>(145.016 – 275.231)	157.728 (39)<br>(113.235 – 217.705)	207.674 (39)<br>(147.726 – 291.948)	154.746 (42)<br>(113.538 – 210.910)	175.293 (81)<br>(142.110 – 223.689)
	t=1.0366, df=79, p=0.3031		t=1.2951, df=79, p=0.1991		
Basophils (%) ** (z, p)	2 (47)	1 (41)	2 (44)	1.25 (44)	1.5 (88)
	z=1.642, p=0.1006		z=1.537, p=0.1242		
Azurophils* $10^3/\mu l$ (t, df, p)	2674.991 (45)<br>(2125.111 – 3300.235)	3203.811 (40)<br>(2594.398 – 3887.311)	2621.282 (42)<br>(2102.446 – 3276.050)	3190.705 (43)<br>(2310.37 – 3872.837)	2921.618 (85)<br>(2509.106 – 3370.769)
	t=-1.2207, df=83, p=0.2257		t=-1.2493, df=83, p=0.2151		
Azurophils (%) (t, df, p)	25.211 (47)<br>(21.356 – 29.408)	26.820 (41)<br>(22.324 – 31.754)	24.691 (44)<br>(20.778 – 28.965)	22.882 (44)<br>(23.033 – 32.025)	25.977 (88)<br>(23.057 – 29.083)
	t=-0.5286, df=86, p=0.5984		t=-0.8427, df=86, p=0.4017		
Lymphocytes $10^3/\mu l$* (t, df, p)	3675.978 (47)<br>(2849.256 – 4742.575)	3964.564 (41)<br>(2353.507 – 5923.396)	3767.136 (44)<br>(2880.344 – 4927.532)	3848.518 (44)<br>(2641.07 – 5607.959)	3807.722 (88)<br>(3035.756 – 4775.996)
	t=-0.3290, df=86, p=0.7429		t=-0.0930, df=86, p=0.9262		
Lymphocytes (%) (t, df, p)	45.245 (47)<br>(40.141 – 50.348)	48.134 (41)<br>(41.284 – 54.984)	45.580 (44)<br>(40.169 – 50.990)	47.602 (44)<br>(41.176 – 54.028)	46.591 (88)<br>(41.284 – 54.984)
	t=-0.6930, df=86, p=0.4902		t=-0.4856, df=86, p=0.6285		
Monocytes $10^3/\mu l$** (z, p)	210 (47)	150 (41)	205 (44)	155 (44)	185 (88)
	z=1.427, p=0.1536		z=1.019, p=0.3084		
Monocytes (%) *	2.403 (45)	1.615 (37)	2.313 (42)	1.732 (40)	2.009 (82)

(t, df, p)	(2.020 – 2.860)	(1.201 – 2.172)	(1.935 – 2.765)	(1.301 – 2.305)	(1.701 – 2.372)
	t=2.439, df=80, **p<0.05**		t=1.7547, df=80, p=0.0831		
Haematocrit (%) (t, df, p)	26.071 (42) (24.436 – 27.707)	27.707 (41) 26.056 – 29.385)	26.308 (39) (24.610 – 28.005)	27.386 (44) (25.772 – 29.001)	26.880 (83) (25.728 – 28.031)
	t=-1.422, df=81, p=0.1588		t=-0.9296, df=81, p=0.3553		
Haemoglobin (g/dl) (t, df, p)	8.000 (47) (7.509 – 8.487)	8.629 (41) (8.019 – 9.239)	8.032 (44) (7.529 – 8.535)	8.552 (44) (7.962 – 9.143)	8.292 (88) (7.908 – 8.676)
	t=-1.6461, df=86, p=0.1034		t=-13533, df=86, p=0.1795		
MCHC g/dl (t, df, p)	30.319 (47) (29.434 – 31.204)	31.244 (41) (30.0117 – 32.476)	30.182 (44) (29.254 – 31.110)	31.318 (44) (31.166 – 32.470)	30.75 (88) (30.152 – 31.485)
	t=-1.2518, df=86, p=0.2140		t=-1.5491, df=86, p=0.1250		

Table S2. Haematological parameters of BIBD and reptarenavirus positive and negative *B. constrictor.*

*Reporting geometric means as data were log transformed, **Wilcoxon rank sum, reporting median because no transformation led to normal distribution

Abbreviations: BIBD: boid inclusion body disease, confirmed by the presence of cytoplasmic inclusion bodies in blood cells (blood smear); CI: confidence intervals; df: degree of freedom; MCHC: mean corpuscular haemoglobin concentration; neg: negative; pos: positive; vRAV: viral RNA (reptarenaviruses).

Table S3. Immune function assays of BIBD and reptarenavirus positive and negative *B. constrictor.*

	**BIBD- (Mean, n, 95%CI)**	**BIBD+ (Mean, n, 95%CI)**	**vRNA- (Mean, n, 95%CI)**	**vRNA+ (Mean, n, 95%CI)**	**Total (Mean, n, 95%CI)**
Iba1+ phagocytic cells % (t, df, p)	64.357 (49) (60.142 – 68.571)	52.202 (33) (45.885 – 58.519)	64.928 (46) (60.533 – 69.322)	52.485 (36) (46.646 – 58.324)	59.465 (82) (55.730 – 63.2)
	t=3.3725, df=80, **p<0.01**		t=3.5151, df=80, **p<0.001**		
LBTT control (t, df, p)	20.268 (33) (16.717 – 23.819)	13.442 (13) (10.800 – 16.084)	19.540 (26) (15.507 – 23.573)	15.061 (15) (11.872 – 18.251)	18.339 (46) (15.584 – 21.094)
	t=2.3581, df=44, **p<0.05**		t=1.5863, df=39, p=0.1207		
LBTT PHA (t, df, p)	21.502 (33) (18.296 – 24.707)	17.803 (13) (13.777 – 21.829)	20.463 (26) (16.870 – 24.057)	18.939 (15) (14.988 – 22.891)	20.457 (46) (17.923 – 22.990)
	t=1.3355, df=44, p=0.1886		t=0.5657, df=39, p=0.5749		
Lysozyme* ug/ml (t, df, p)	2.124 (44) (1.819 – 3.524)	1.879 (35) (1.615 – 2.185)	2.019 (43) (1.728 – 2.359)	2.003 (36) (1.646 – 2.436)	2.0112 (79) (1.784 – 2.268)
	t=1.010, df=77, p=0.3158		t=0.0665, df=77, p=0.9472		
HA Sheep** (z, p)	6 (44)	6 (35	6 (42)	6 (36)	6 (79)
	z=-0.239, p=0.8108		z=-0.620, p=0.5353		
HL Sheep** (z, p)	5 (44)	5 (35)	5 (42)	5 (36)	5 (79)
	z=0.368, p=0.7130		z=-0.016, p=0.9876		
Total immunoglobulins* mg/ml (t, df,p)	16.971 (55) (14.670 – 19.633)	11.229 (35) (9.271 – 13.6001)	17.062 (51) (14.649 – 19.873)	11.673 (38) (9.689 – 14.078)	14.453 (90) (12.500 – 16.319)
	t=3.4959, df=88, **p<0.001**		t=3.1958, df=87, **p<0.01**		
UGV-1 IgY OD values** (z, p)	0.0528 (60) (0.04-0.089)	0.222 (35) (0.17-0.36)	0.0528 (60) (0.04-0.089)	0.222 (36) (0.17-0.36)	0.092 (96) (0.062-0.17)
	z= -4.1, **p<0.0001**		z= -3.89, **p<0.0001**		
UGV-1 IgM OD values** (z, p)	0.0691 (60) (0.057-0.081)	0.125 (35) (0.098-0.149)	0.0691 (60) (0.057-0.081)	0.125 (36) (0.098-0.149)	0.082 (96) (0.069-0.11)
	-3.75, **p<0.0001**		z =-3.68, **p= 0.0002**		

*Reporting geometric means as data were log transformed, **Wilcoxon rank sum, reporting median because no transformation led to normal distribution

Abbreviations: BIBD: boid inclusion body disease, confirmed by the presence of cytoplasmic inclusion bodies in blood cells (blood smear); CI: confidence intervals; df: degree of freedom; HA: haemagglutination; HL: haemolysis; Iba1: ionized calcium binding adaptor molecule 1; IgM: immunoglobulin M; IgY: immunoglobulin Y; LBTT: leukocyte blast transformation test; neg: negative; OD: optical density; PHA: phytohaemagglutinin; pos: positive; UGV-1: university of Giessen virus-1; vRAV: viral RNA (reptarenaviruses).

Table S4. Biochemical data and physiological stress parameters of BIBD and reptarenavirus positive and negative *B. constrictor*.

	**BIBD- (Mean, n, 95%CI)**	**BIBD+ (Mean, n, 95%CI)**	**vRNA- (Mean, n, 95%CI)**	**vRNA+ (Mean, n, 95%CI)**	**Total (Mean, n, 95%CI)**
Cholesterol* mmol/l (t, df, p)	2.293 (73) (1.969 – 2.671)	1.654 (41) (1.301 – 2.103)	2.381 (64) (2.015 – 2.814)	1.680 (44) (1.336 – 2.113)	2.039 (114) (1.788 – 2.325)
	t=2.4122, df=112, **p<0.05**		t=2.5261, df=106, **p<0.05**		
Urea* mmol/l (t, df, p)	0.204 (63) (0.168 – 0.248)	0.125 (29) (0.081 – 0.194)	0.202 (56) (0.164 – 0.249)	0.140 (31) (0.095 – 0.205)	0.175 (92) (0.144 – 0.212)
	t=2.4115, df=90, p<0.05		t=1.8733, df=85, p=0.645		
Total protein g/l (t, df, p)	47.724 (73) (44.207 – 50.648)	50.263 (41) (46.024 – 54.503)	48.633 (64) (12.3.2 – 51.631)	50.445 (44) (46.334 – 54.557)	48.639 (114) (46.255 – 51.024)
	t=-1.0112, df=112, p=0.3141		t=-0.7322, df=106, p=0.4657		
AST, U/i* (t, df, p)	19.181 (73) 16.105 – 22.844)	23.032 (41) (17.478 – 30.352)	19.191 (64) (15.8363 – 23.218)	24.019 (44) (18.467 – 31.240)	20.486 (114) (17.668 – 23.753)
	t=-1.1779, df=112, p=0.2413		t=-1.4223, df=106, p=0.1579		
Albumin, g/l (t, df, p)	26.804 (73) (25.249 – 28.360)	27.905 (41) (25.612 – 30.198)	27.242 (64) (25.595 – 28.890)	27.916 (44) (25.779 – 30.052)	27.2 (114) 25.924 – 28.476)
	t=-0.819, df=112, p=0.4145		t=-0.5079, df=106, p=0.6126		
LDH (U/l) * (t, df, p)	106.211 (73) (88.612 – 127.235)	94.941 (41) (73.297 – 122.977)	106.583 (64) (87.616 – 129.655)	101.014 (44) (78.572 – 129.867)	102.002 (114) (88.118 – 118.096)
	t=0.7269, df=112, p=0.4688		t=0.3417, df=106, p=0.7332		
Uric acid* mmol/l (t, df, p)	0.2001 (73) (0.178 – 0.226)	0.233 (41) (0.178 – 0.274)	0.206 (64) (0.181 – 0.234)	0.233 (44) (0.199 – 0.273)	0.212 (114) (0.192 – 0.233)
	t=-1.458, df=112, p=0.1476		t=-1.2307, df=106, p=0.2212		
Corticosterone* ng/ml (t, df, p)	4.493 (35) (3.121 – 4.468)	7.484 (32) (4.442 – 12.611)	4.462 (25) (3.155 – 6.310)	8.309 (34) (4.863 – 14.109)	5.733 (67) (4.199 – 7.827)
	t=---1.6558, df=65, p=0.1026		t=-1.8485, df=57, p=0.0697		
Glucose mmol/l (t, df, p)	5.499 (40) (3.824 – 7.477)	5.901 (41) (4.550 – 7.43)	2.119 (37) (1.754 – 2.519)	2.392 (44) (2.108 – 2.693)	5.701 (81) (4.618 – 7.427)
	t=-0.3492, df=79, p=0.7278		t=-1.1587, df=79, p=0.2501		

Heterophil/lymphocyte ratio<br>(t, df, p)	0.005 (47)<br>(0.003 – 0.007)	0.004 (41)<br>(0.002 – 0.006)	0.005 (44)<br>(0.003 – 0.007)	0.004 (44)<br>(0.002 – 0.006)	0.004 (88)<br>(0.003 –0.006)
	t=0.9989, df=86, p=0.3207		t=0.8056, df=86, p=0.4227		

*Reporting geometric means as data were log transformed

<u>Abbreviations:</u> AST: aspartate aminotransferase; BIBD: boid inclusion body disease, confirmed by the presence of cytoplasmic inclusion bodies in blood cells (blood smear); CI: confidence intervals; df: degree of freedom; LDH: lactate dehydrogenase; neg: negative; pos: positive; vRAV: viral RNA (reptarenaviruses).

Table S5. Regression analysis of viral infection, age, and sex against haematological parameters, biochemical data, physiological stress parameters, and immune function parameters in *B. constrictor*

	**Reptarenavirus**			**Age (square root)**			**Sex**			**Constant**			**Model**
	Coef	95%CI	p	Coef	95%CI	p	Coef	95%CI	p	Coef	95%CI	p	
Leukocytes (10^3/µl) n=72	0.043	-0.084-0.170	0.427	-0.058	-0.186-0.069	0.294	-0.017	-0.227-0.192	0.840	4.117	3.868-4.367	<0.001	F(3,5)=1.83, R^2=0.0382 p=0.2596 (6 clusters)
Heterophils (%) n=72	-0.111	-0.252-0.030	0.098	0.130	0.066-0.255	**<0.01**	0.054	-0.133-0.241	0.490	0.870	0.542-1.199	<0.01	F(3,5)=34.39, R^2=0.0.2460 p<0.001 (6 clusters)
Heterophils (10^3/µl) n=70	-0.093	-0.163--0.024	**<0.05**	0.119	0.002-0.236	**<0.05**	0.039	-0.047-0.185	0.187	2.950	2.679-3.221	<0.001	F(3,5)=188.86, R^2=0.1867 p<0.001 (6 clusters)
Eosinophils (%) n=72	0.192	-0.646-1.030	0.581	-0.193	-0.444-0.058	0.105	-0.160	-0.572-0.058	0.363	1.575	0.210-2.940	<0.05	F(3,5)=55.18, R^2=0.0308 p<0.001 (6 clusters)
Eosinophils (10^3/µl) n=72	-19.556	-167.26-128.15	**<0.01**	-57.233	-78.93--35.537	**<0.01**	-87.558	207.89-29.77	0.113	332.26	119.45-547.07	<0.05	F(3,5)=27.99, R^2=0.0733 p<0.01 (6 clusters)
Basophils (%) n=72	-0.470	-3.976-3.036	0.745	-0.143	-1.141-0.855	0.728	0.205	-0.852-1.262	0.639	2.823	1.424-4.222	<0.01	F(3,5)=0.58, R^2=0.0156 p=0.6545 (6 clusters)
Basophils (10^3/µl) n=65	-0.090	-0.584-0.405	0.661	-0.115	-0.238-0.009	0.063	-0.066	-0.323-0.190	0.535	2.556	2.227-2.885	<0.001	F(3,5)=4.96, R^2=0.0726 p=0.0585 (6 clusters)
Azurophils (%) n=72	0.053	-1.195-1.301	0.917	0.456	0.202-0.709	**<0.01**	0.173	-0.866-1.212	0.686	3.829	2.289-5.70	<0.01	F(3,5)=8.72, R^2=0.0820 p<0.05 (6 clusters)
Azurophils (10^3/µl) n=69	2.499	-12.704-17.702	0.690	3.232	-4.298-10.762	0.320	2.612	-7.670-12.893	0.543	41.714	20.948-62.480	<0.01	F(3,5)=0.54, R^2=0.0280 P=0.6779 (6 clusters)
Lymphocytes (%) n=72	2.693	-12.921-18.306	0.676	-10.181	-14248--6.115	**<0.01**	-1.772	-20.037-16.493	0.813	69.746	43.898-95.594	<0.01	F(3,5)=19.44, R^2=0.2110 p<0.01 (6 clusters)
Lymphocytes (10^3/µl) n=72	0.116	-0.227-0.250	0.906	-0.165	-0.313--0.017	**<0.05**	0.042	-0.400--0.017	0.818	3.948	3.575-4.322	<0.001	F(3,5)=3.42, R^2=0.1078 P=0.1094 (6 clusters)

Monocytes (%) n=72	0.357	-2.278-2.349	0.970	0.115	-0.946-1.145	0.786	-0.124	-1.822-1.574	0.859	2.151	-0.019-4.320	0.051	F(3,5)=0.03, R^2=0.0021 p<0.9905 (6 clusters)
Monocytes (10^3/µl) n=72	140.289	26.2001-224.37	**<0.01**	-195.340	-112.98-52.30	0.098	-129.93	-633.37-373.50	0.536	747.60	64.450-1430.76	<0.05	F(3,5)=6.95, R^2=0.0438 p<0.05 (6 clusters)
Haematocrit n=67	-0.749	-1.725-0.228	0.106	1.156	0.317-1.995	**<0.05**	-1.047	-5.874-3.780	0.601	36.395	23.108-29.682	<0.001	F(3,5)=10.99, R^2=0.0828 p<0.05 (6 clusters)
Haemoglobin (g/dl) n=72	0.157	-0.208-0.522	0.319	0.363	-0.417-1.143	0.285	-0.122	-1.959-1.716	0.872	7.754	5.437-10.072	<0.001	F(3,5)=23.20, R^2=0.0455 p<0.01 (6 clusters)
MCHC n=72	0.024	0.003-0.045	**<0.05**	-0.002	-0.023-0.019	0.827	0.008	-0.009-0.25	0.259	1.469	1.420-1.518	<0.001	F(3,5)=65.08, R^2=0.0520, p<0.001 (6 clusters)
Cholesterol, mmol/l, n=88	-0.179	-0.313--0.045	**<0.05**	0.130	0.092-0.247	**<0.01**	0.130	0.057-0.203	**<0.01**	-0.060	-0.220-0.100	0.420	F(3,9)=27.03, R^2=0.2786 p<0.001 (10 clusters)
Urea mmol/l, n=73	-0.054	-0.176-0.068	0.344	0.029	-0.062-0.120	0.493	0.015	-0.099-0.129	0.769	0.202	-0.014-0.418	0.063	F(3,9)=0.54, R^2=0.0168 p=0.6643 (10 clusters)
Total protein g/l, n=88	-1.0807	-7.678-4.064	0.504	7.905	6.266-9.543	**<0.001**	-1.378	-6.361-3.606	0.547	34.239	24.051-44.428	<0.001	F(3,9)=55.84, R^2=0.2990 p<0.001 (10 clusters)S
AST U/i , n=88	0.071	-0.051-0.192	0.223	0.039	-0.162-0.240	0.223	0.147	0.009-0.285	**<0.05**	1.145	0.009-0.285	<0.05	F(3,9)=3.98, R^2=0.051 p<0.05 (10 clusters)
Albumin g/l, n=88	-1.618	-4.026-0.790	0.163	4.041	2.864-5.218	**<0.001**	0.254	-2.170-2.677	0.818	19.804	14.730-24.878	<0.001	F(3,9)=22.71, R^2=0.2432 p<0.001 (10 clusters)
LDH U/l, n=88	-0.042	-0.182-0.099	0.517	0.023	-0.158-0.203	0.784	0.119	-0.047-0.286	0.139	1.933	1.525-2.340	<0.001	F(3,9)=1.07, R^2=0.0323 p=0.4091 (10 clusters)
Uric acid mmol/l, n=88	0.034	-0.151-0.218	0.691	0.051	-0.040-0.143	0.238	0.261	-0.082-0.135	0.599	-0.810	-1.147--0.472	<0.001	F(3,9)=0.63, R^2=0.0463 p=0.6128 (10 clusters)
Glucose mmol/l, n=67	0.094	-0.098-0.286	0.263	-0.026	-0.171-0.119	0.669	-0.075	-0.184-0.034	0.136	1.548	1.298-1.798	<0.001	F(3,5)=3.55, R^2=0.0333 p=1.030 (6 clusters)
Corticosterone ng/ml, n=53	0.293	-0.329-0.916	0.302	0.089	-0.267-0.444	0.575	-0.260	-0.602-0.083	0.116	0.522	-0.265-0.083	0.161	F(3,7)=1.4, R^2=0.1327 p=0.3166 (8 clusters)
Heterophil/Lymphocyte ratio, n=72	-0.123	-0.496-0.251	0.436	0.325	0.132-0.518	**<0.01**	0.012	-0.585-0.610	0.959	-3.078	-3.773-2.383	<0.001	F(3,5)=1.4, R^2=0.1327 p=0.3166 (6 clusters)
Iba1+ phagocytic cells, n=65	-14.305	-21.217--7.393	**<0.01**	2.398	-3.046-7.830	0.323	-8.289	-20.888-4.310	0.159	65.405	49.457-4.3009	<0.001	F(3,5)=61.08, R^2=0.2489 p<0.001 (7 clusters)

LBTT control n=32*	-7.395	13.643--1.146	**<0.05**	-1.969	-6.543-2.605	0.333	1.546	-3.247-6.339	0.460	27.380	12.384-42.376	<0.01	F(3,6)=6.49, R^2=0.2335 p<0.05 (7 clusters)
LBTT PHA n=32	-3.083	-10.254-40.089	0.333	-1.500	-6.261-3.260	0.470	3.387	-2.256-9.029	0.192	24.338	13.648-35.027	<0.01	F(3,6)=3.41, R^2=0.1004 p=0.0937 (7 clusters)
Lysozyme n=64	0.010	-0.186-0.205	0.911	0.99	0.027-0.172	**<0.05**	-0.066	-0.130-0.002	**<0.05**	0.106	-0.115-0.327	0.295	F(3,7)=6.77, R^2=0.1501 p<0.5 (8 clusters)
HA Sheep n=64	-0.195	-0.778-0.389	0.445	0.560	-0.231-1.430	0.128	0.183	-0.460-0.826	0.513	4.945	-0.460-6.325	<0.001	F(3,6)=1.11, R^2=0.115 p=0.3224 (7 clusters)
HL Sheep n=64	-0.308	-0.906-0.289	0.254	0.545	0.095-0.996	**<0.05**	0.104	-0.646-0.855	0.104	3.896	2.977-43815	0.746	F(3,6)=3.87, R^2=0.163 p=0.0747 (6 clusters)
Total Immunoglobulins n=78*	-6.925	-11.744--2.107	**<0.05**	1.099	-0.939-3.136	0.249	-1.257	-9.690-7.176	0.740	17.760	14.004-21.516	<0.001	F(3,8)=13.63, R^2=0.0893 p<0.01 (9 clusters)

Abbreviations: AST: aspartate aminotransferase; BIBD: boid inclusion body disease, confirmed by the presence of cytoplasmic inclusion bodies in blood cells (blood smear); CI: confidence intervals; df: degree of freedom; HA: haemagglutination; HL: haemolysis; Iba1: ionized calcium binding adaptor molecule 1; LBTT: leukocyte blast transformation test; LDH: lactate dehydrogenase; MCHC: mean corpuscular haemoglobin concentration; neg: negative; PHA: phytohaemagglutinin; pos: positive; vRAV: viral RNA (reptarenaviruses).

Table S6. Regression analysis of BIBD, age, and sex against haematological parameters, biochemical data, physiological stress parameters, and immune function parameters in *B. constrictor*

	**BIBD**			**Age (square root)**			**Sex**			**Constant**			**Model**
	Coef	95%CI	p	Coef	95%CI	p	Coef	95%CI	p	Coef	95%CI	p	
Leukocytes 10^3/μl	0.668	-0.069-0.203	0.262	-0.058	-0.181-0.065	0.282	-0.017	-0.224-0.191	0.846	4.103	3.858-4.348	<0.001	F(3,5)=6.42, R^2=0.046 p<0.05
Heterophils %	-0.120	-0.267-0.026	0.088	0.159	.061-0.256	**<0.01**	0.056	-0.127-0.238	0.469	0.877	0.534-1.218	<0.01	F(3,5)=53.42, R^2=0.2519, p<0.001
Heterophils 10^3/μl	-0.078	-0.131-0.024	**<0.05**	0.118	-0.000-0.235	0.050	0.071	-0.052-0.194	0.198	2.943	2.691-3.194	<0.001	F(3,5)=161.04, R^2=0.1778, p<0.001
Eosinophils %	0.033	-0.217-0.284	0.746	-0.039	-0.096-0.019	0.144	0.236	-0.196-0.121	0.571	0.236	0.058-0.414	<0.05	F(3,5)=1.75, R^2=0.0255, p=0.2729
Eosinophils 10^3/μl	0.068	-0.231-0.367	0.584	-0.102	-0.252-0.048	0.140	-0.127	-0.292-0.038	0.105	2.428	2.111-2.744	<0.001	F(3,5)=1.95, R^2=0.0.0917, p=0.2403
Basophils %	-0.517	-3.973-2.938	0.716	-0.150	-1.086-0.786	0.697	0.210	1.433-1.227	0.618	2.857	2.857-0.554	<0.01	F(3,5)=0.63, R^2=0.176, p=0.6252
Basophils 10^3/μl	-0.081	-0.551-0.389	0.678	-0.116	-0.239-0.008	0.061	-0.064	-0.327-0.199	0.561	2.551	2.231-2.870	<0.001	F(3,5)=4.51, R^2=0.0706, p=0.0706
Azurophils %	0.120	-0.958-1.199	0.786	0.123	0.195-0.171	**<0.01**	0.177	-0.850-1.203	0.677	3.790	2.338-5.243	<0.01	F(3,5)=8.57, R^2=0.0834, p<0.05
Azurophils 10^3/μl	4.238	-6.757-15.234	0.367	3.221	-4.05-10.493	0.367	2.641	-7.962-13.244	0.550	40.778	21.9236-59.633	<0.01	F(3,5)=1.17, R^2=0.0355, p=0.4092
Lymphocytes %	2.472	-11.960-16.903	0.678	-10.138	-14.327-5.950	**<0.01**	-1.836	-19.705-16.032	0.802	69.838	44.560-95.11	<0.01	F(3,5)=22.38, R^2=0.2104, p<0.001
Lymphocytes 10^3/μl	0.268	-0.206-0.260	0.779	-0.165	-0.309-0.020	**<0.05**	0.043	-0.396-0.481	0.813	3.939	3.558-4.320	<0.001	F(3,5)=5.31, R^2=0.1084, p=0.0518
Monocytes %	-0.131	-2.32-1.969	0.879	0.116	-0.905-1.137	0.782	-0.136	-1.915-1.643	0.852	2.247	0.279-4.214	<0.05	F(3,5)=0.08, R^2=0.0026, p=0.0518
Monocytes 10^3/μl	139.013	59.601-218.43	**<0.01**	-193.13	-442.45-56.21	0.103	-132.58	-634.15-368.99	0.527	746.49	60.240-1432.75	<0.05	F(3,5)=7.96, R^2=0.0437, p=0.9706
Haematocrit	-0.690	-1.695-0.314	0.138	1.145	0.328-1.962	**<0.05**	-1.029	-5.893-3.834	0.610	26.367	23.129-29.605	<0.001	F(3,5)=11.92, R^2=0.0820, p<0.04
Haemoglobin	0.107	-0.304-0.519	0.533	0.366	-0.421-1.152	0.285	-0.128	-1.951-1.695	0.864	7.781	5.452-10.110	<0.001	F(3,5)=23.82, R^2=0.0444, p<0.05
MCHC	0.020	-0.05-0.046	0.096	-0.002	-0.023-0.020	0.856	0.008	-0.007-0.022	0.226	1.471	1.420-1.521	<0.001	F(3,5)=38.52, R^2=0.0401, p<0.001

Cholesterol	-0.151	-0.277--0.024	**<0.05**	0.156	0.083-0.229	**<0.01**	0.130	0.065-0.196	**<0.01**	-0.052	-0.209-0.104	0.468	$F(3,9)=43.32$, $R^2=0.2370$, $p<0.001$
Urea	0.089	-0.179-0.002	0.054	0.047	-0.001-0.095	0.055	0.029	-0.082-0.140	0.570	0.149	0.015-0.282	<0.05	$F(3,9)=2.44$, $R^2=0.0722$, $p=0.1317$
Total protein	-0.585	-6.507-5.337	0.828	7.751	5.384-10.118	**<0.001**	-0.553	-5.869-4.762	0.819	32.663	20.585-44.741	<0.001	$F(3,9)=47.36$, $R^2=0.2573$, $p<0.001$
AST	0.085	-0.048-0.217	0.184	0.356	-0.157-0.228	0.686	0.151	0.114-0.291	**<0.05**	1.134	0.722-1.547	<0.001	$F(3,9)=5.68$, $R^2=0.0551$, $p<0.05$
Albumin	-0.6222	-3.314-2.070	0.614	3.975	2.612-5.299	**<0.001**	0.791	-1.680-3.262	0.487	18.780	12.825-24.736	<0.001	$F(3,9)=19.80$, $R^2=0.2325$, $p<0.001$
LDH	-0.044	-0.178-0.091	0.482	0.017	-0.119-0.091	0.788	0.129	-0.031-0.289	0.101	1.928	1.582-2.274	<0.001	$F(3,9)=1.12$, $R^2=0.0393$, $p=0.3909$
Uric acid	0.067	-0.144-0.278	0.490	0.051	-0.043-0.145	0.253	0.025	-0.077-0.126	0.596	-0.831	-1.190--0.472	<0.01	$F(3,9)=0.68$, $R^2=0.0327$, $p=0.5863$
Glucose	0.367	-0.067-0.140	0.403	-0.024	-0.156-0.109	0.663	-0.084	-0.196-0.209	0.114	1.583	1.378-1.789	<0.001	$F(3,5)=4.94$, $R^2=0.0190$, $p=0.0588$
Corticosterone	0.224	-0.348-0.796	0.385	0.080	-0.281-0.442	0.615	-0.227	-0.499-0.045	0.089	0.543	-0.167-1.253	0.113	$F(3,7)=1.66$, $R^2=0.1049$, $p=0.2606$
Heterophil/lymphocyte ratio	-0.147	-0.521-0.226	0.357	0.323	0.133-0.514	**<0.01**	0.013	-0.575-0.601	0.957	-3.062	-3.777--2.348	<0.001	$F(3,5)=22.01$, $R^2=0.1775$, $p<0.01$
Phagocytic Iba1+ cells	-14.406	-21.017--7.795	**<0.01**	2.110	-3.550-7.771	0.397	-8.039	-20.289-4.211	0.159	65.772	49.515-82.028	<0.001	$F(3,6)=83.22$, $R^2=0.2503$, $p<0.001$
LBTT Control	-8.414	-12.888--3.940	**<0.01**	-1.710	-5.465-2.046	0.308	0.922	-3.735-5.579	0.645	26.499	14.453-38.546	<0.01	$F(3,6)=5.31$, $R^2=0.2600$, $p<0.05$
LBTT PHA	-3.957	-8.567-0.652	0.080	-1.298	-5.528-2.931	0.481	3.091	-2.433-8.616	0.220	23.988	14.875-33.101	<0.01	$F(3,6)=6.11$, $R^2=0.1154$, $p<0.05$
Lysozyme	-0.048	-0.122-0.025	0.164	0.089	0.021-0.157	**<0.05**	-0.071	-0.160-0.018	0.103	0.164	0.014-0.313	<0.05	$F(3,7)=9.69$, $R^2=0.1593$, $p<0.1$
HA Sheep	-0.409	-0.837-0.020	0.058	0.570	-0.198-1.338	0.120	0.163	-0.546-0.873	0.594	5.136	4.065-6.207	<0.001	$F(3,6)=1.86$, $R^2=0.1278$, $p=0.2376$
HL Sheep	-0.418	-1.119-0.283	0.195	0.518	0.101-0.934	**<0.05**	0.109	-0.651-0.869	0.738	4.016	3.178-4.858	<0.001	$F(3,6)=4.94$, $R^2=0.1729$, $p<0.05$
Total immunoglobulins	-7.348	-12.653--2.044	**<0.05**	1.235	-0.740-3.210	0.187	-1.611	-9.743-6.522	0.660	17.606	13.701-21.512	<0.001	$F(3,5)=6.69$, $R^2=0.0999$, $p<0.05$

Abbreviations: AST: aspartate aminotransferase; BIBD: boid inclusion body disease, confirmed by the presence of cytoplasmic inclusion bodies in blood cells (blood smear); CI: confidence intervals; df: degree of freedom; HA: haemagglutination; HL: haemolysis; Iba1: ionized calcium binding adaptor molecule 1; LBTT: leukocyte blast transformation test; LDH: lactate dehydrogenase; MCHC: mean corpuscular haemoglobin concentration; neg: negative; PHA: phytohaemagglutinin; pos: positive; vRAV: viral RNA (reptarenaviruses).

Table S7. Kruskal–Wallis and Dunn’s multiple comparisons analysis of parameters in BIBD−/vRNA−snakes , BIBD+/vRNA+ snakes without clinical sings and BIBD+/vRNA+ snakes with clinical signs.

Parameter	Kruskal–Wallis H	Overall adjusted p	BIBD-/vRNA- (n=82) vs. BIBD+/vRNA-+ with clinical signs (n=5)	BIBD+/vRNA-+ without clinical signs (n=43) vs. BIBD+/vRNA-+ with clinical signs (n=5)
Leukocytes (10^3/µl)	0.91	0.63	>0.99	>0.99
Heterophils (%)	2.71	0.26	>0.99	>0.99
Heterophils (10^3//µl)	1.05	0.59	>0.99	>0.99
Eosinophils (%)	0.14	0.93	>0.99	>0.99
Eosinophils (10^3//µl)	0.45	0.80	>0.99	>0.99
Basophils (%)	2.63	0.27	>0.99	>0.99
Basophils (10^3//µl)	1.07	0.58	>0.99	>0.99
Azurophils (%)	0.09	0.96	>0.99	>0.99
Azurophils (10^3//µl)	1.66	0.44	>0.99	>0.99
Lymphocytes (%)	0.75	0.69	>0.99	>0.99
Lymphocytes (10^3//µl)	0.31	0.86	>0.99	>0.99
Monocytes (%)	8.98	0.01	0.18	>0.99
Monocytes (10^3//µl)	2.48	0.29	0.64	>0.99
Heamatocrit	2.37	0.31	0.41	0.79
Haemoglobin (g/dl)	5.12	0.08	0.09	0.28
MCHC	6.59	0.04	0.09	0.58
Cholesterol	4.26	0.12	>0.99	>0.99
Urea	4.93	0.09	>0.99	>0.99
Total protein	2.69	0.26	0.30	0.43
AST	3.04	0.22	0.25	0.42
Albumin	3.70	0.16	0.17	0.2
LDH	2.02	0.36	>0.99	0.72

Uric acid	0.90	0.64	>0.99	>0.99
Glucose	0.69	0.71	>0.99	>0.99
Corticosterone	2.82	0.24	>0.99	>0.99
Heterophil/lymphocyte ratio	1.04	0.60	>0.99	>0.99
Iba1+ phagocytic cells	9.19	0.01	0.09	>0.99
Lysozyme	0.82	0.66	>0.99	>0.99
HA Sheep	0.55	0.76	>0.99	>0.99
HL Sheep	0.14	0.93	>0.99	>0.99
Total immunglobulins	10.75	0.00	0.19	>0.99
UGV-1 IgY OD	2.21	0.33	0.67	>0.99
UGV-1 IgM OD	13.35	0.00	0.06	>0.99

Abbreviations: AST: aspartate aminotransferase; BIBD: boid inclusion body disease, confirmed by the presence of cytoplasmic inclusion bodies in blood cells (blood smear); HA: haemagglutination; HL: haemolysis; Iba1: ionized calcium binding adaptor molecule 1; LBTT: leukocyte blast transformation test; LDH: lactate dehydrogenase; MCHC: mean corpuscular haemoglobin concentration; vRAV: viral RNA (reptarenaviruses).
